# Hybrid Adaptive Crayfish Optimization with Differential Evolution for Color Multi-Threshold Image Segmentation

**DOI:** 10.3390/biomimetics10040218

**Published:** 2025-04-02

**Authors:** Honghua Rao, Heming Jia, Xinyao Zhang, Laith Abualigah

**Affiliations:** 1 School of Electrical and Information Engineering, Northeast Petroleum University, Daqing 163318, China; raohonghua@stu.nepu.edu.cn; 2 School of Information Engineering, Sanming University, Sanming 365004, China; 3 School of Business, Jinggangshan University, Jian 343000, China; 2104101029@jgsu.edu.cn; 4 Computer Science Department, Al al-Bayt University, Mafrag 25113, Jordan; aligah@ammanu.edu.jo

**Keywords:** color multi-threshold image segmentation, crayfish optimization algorithm, adaptive foraging quantity adjustment strategy, Kapur entropy method, Otsu method

## Abstract

To better address the issue of multi-threshold image segmentation, this paper proposes a hybrid adaptive crayfish optimization algorithm with differential evolution for color multi-threshold image segmentation (ACOADE). Due to the insufficient convergence ability of the crayfish optimization algorithm in later stages, it is challenging to find a more optimal solution for optimization. ACOADE optimizes the maximum foraging quantity parameter *p* and introduces an adaptive foraging quantity adjustment strategy to enhance the randomness of the algorithm. Furthermore, the core formula of the differential evolution (DE) algorithm is incorporated to balance ACOADE’s exploration and exploitation capabilities better. To validate the optimization performance of ACOADE, the IEEE CEC2020 test function was selected for experimentation, and eight other algorithms were chosen for comparison. To verify the effectiveness of ACOADE for threshold image segmentation, the Kapur entropy method and Otsu method were used as objective functions for image segmentation and compared with eight other algorithms. Subsequently, the peak signal-to-noise ratio (PSNR), feature similarity index measure (FSIM), structural similarity index measure (SSIM), and Wilcoxon test were employed to evaluate the quality of the segmented images. The results indicated that ACOADE exhibited significant advantages in terms of objective function value, image quality metrics, convergence, and robustness.

## 1. Introduction

Image segmentation is a crucial research direction in the field of computer vision, aiming to partition images into multiple regions with similar properties or meanings for further analysis and processing [1]. With the continuous advancement of digital technology, image segmentation has demonstrated extensive application prospects in numerous fields, including medical image analysis [2], autonomous driving [3], intelligent monitoring [4], and remote sensing image processing [5]. Compared with grayscale images, color images exhibit significant advantages in visual perception, reversible information hiding, and technological applications, making them widely utilized and valued in various domains [6,7,8].

The existing image segmentation methods are mainly divided into the following categories: threshold-based segmentation methods [9], color-based methods [10], edge-based segmentation methods [11], clustering-based segmentation methods [12], region-based segmentation methods [13], etc. Among them, threshold-based methods are widely used due to their simple algorithm implementation and good segmentation results. The existing image threshold segmentation algorithms include two classic algorithms: the Kapur entropy method and the Otsu method. The Kapur entropy method is a threshold selection method based on information theory. The basic idea is to select the optimal threshold by calculating the entropy of pixels at different grayscale levels in the image. In an image, the grayscale values of pixels are considered random variables, and the distribution of pixel values can reflect the information content of the image. By calculating the entropy of pixels at different grayscale levels, the Kapur entropy method can find an optimal threshold that maximizes the information difference between segmented foreground and background pixels [14]. Therefore, the Kapur entropy method can serve as an objective function to optimize the effectiveness of image segmentation. This method has a wide range of applications in the field of image processing, especially in scenes that require precise image segmentation [15]. The Otsu method is a widely used image segmentation algorithm [16]. Its core idea is to determine a threshold based on the grayscale characteristics of the image, dividing it into two parts: a set of pixels that meet specific threshold conditions and a set of pixels that do not meet specific threshold conditions. In this process, the Otsu method aims to minimize the intra-class variance by finding a threshold that minimizes the difference between the two segmented pixel sets. This difference is quantified by calculating the grayscale distribution and intra-class variance of pixels. By continuously updating the threshold and recalculating the intra-class variance, the threshold that minimizes the intra-class variance is ultimately found, which is the optimal segmentation or binarization threshold. This method performs well in image segmentation, especially suitable for scenes that require precise differentiation between foreground and background [17]. The Kapur entropy method and Otsu method have been widely applied in image processing after years of in-depth research and practice [18,19,20]. However, these two methods have also exhibited some shortcomings in their applications. The Kapur entropy method, due to its relatively complex calculation process, may result in longer processing time, especially when dealing with large-scale images with lower efficiency. In addition, for certain images with complex textures or noise interference, the Kapur entropy method may be difficult to use to accurately identify the boundaries of different regions, resulting in less accurate segmentation results. The Otsu method may find it difficult to accurately identify the optimal segmentation threshold when processing images with bimodal or multimodal grayscale distributions, resulting in inaccurate segmentation results [21,22,23]. Furthermore, when there is noise or uneven lighting interference in the image, the segmentation effect of the Otsu method may also be affected [24].

To overcome these shortcomings, researchers have proposed a method that combines metaheuristic optimization algorithms to enhance the segmentation performance of the algorithm [25]. Metaheuristic optimization algorithms are a type of universal problem solver inspired by mechanisms such as biological evolution, swarm intelligence, and natural physical and chemical processes. This algorithm type boasts advantages such as robust global search capabilities, fast convergence speed, and relatively straightforward parameter settings. It can iteratively search for the optimal solution to the problem without requiring prior strict mathematical modeling of the problem structure. Therefore, it exhibits significant advantages in solving complex optimization problems. In recent years, numerous metaheuristic optimization algorithms have been employed for image segmentation [26]. For instance, Qingxin Liu proposed the chimpanzee-inspired remora optimization algorithm (HCROA) in 2023. HCROA improves the optimization effect of the remora optimization algorithm and achieves promising results in color multi-threshold image segmentation [27]. Guoyuan Ma presented an enhanced whale optimization algorithm for multi-threshold image segmentation in 2022, addressing the shortcomings of the traditional whale optimization algorithm (WOA) and enhancing the quality and stability of segmentation results in color multi-threshold image segmentation [19].

In 2023, Heming Jia et al. proposed a crayfish optimization algorithm (COA) [28]. This algorithm mainly simulates the foraging, summer avoidance, and competitive behavior of crayfish. The COA has fast search speed and strong search ability, which can effectively balance the ability of global search and local search. The COA is a promising new optimization algorithm. However, there are still problems, such as insufficient convergence ability in the later stage and susceptibility to falling into global optima. Yi Zhang et al. proposed an improved crawfish optimization algorithm, which effectively improved the optimization effect of the algorithm [29]. Nabila H. Shikoun proposed a new binary crayfish optimization algorithm (BinCOA) in 2024, which adds a crisscrossing strategy to the original COA and improves the convergence accuracy of the COA [30].

To better solve the problem of image segmentation, this article proposes a hybrid adaptive crayfish optimization algorithm with differential evolution for color multi-threshold image segmentation (ACOADE). This method significantly improves the flexibility and efficiency of the algorithm in exploring the solution space by finely adjusting the key parameters in the crayfish optimization algorithm, especially the maximum foraging amount p, and introducing an adaptive foraging amount adjustment strategy. Meanwhile, in order to balance the exploration and exploitation capabilities of the algorithm, we cleverly incorporated the core formula of the differential evolution algorithm, ensuring that ACOADE can balance global and local searches when searching for the optimal threshold. To verify the optimization performance of ACOADE, we selected the IEEE CEC2020 test function as the benchmark and conducted in-depth comparisons with eight algorithms. The experimental results showed that ACOADE showed excellent superiority in optimization problems, achieving significant advantages in both solution quality and convergence speed. In addition, in order to verify the practical application effect of ACOADE in image segmentation, we used the classical Kapur entropy method and Otsu method as the objective function of RGB primary color and carried out segmentation experiments on multiple images. To verify the optimization effect of ACOADE, we comprehensively and objectively evaluated the segmentation results using evaluation metrics such as the peak signal noise ratio (PSNR), the feature similarity index (FSIM), and the structural similarity index (SSIM). The experimental results showed that ACOADE had significant advantages in objective function value, image quality measurement, convergence, and robustness. The results fully proved the effectiveness and practicability of ACOADE in the field of color multi-threshold image segmentation.

## 2. Related Work

This section describes the related work. Table 1 shows that different researchers use different methods to solve the problem of image segmentation. They use different test methods to solve the problem of image segmentation and improve the algorithm to enhance the effect. These works show that various improved versions of meta heuristic optimization algorithms are proposed and combined with traditional methods to obtain the optimal threshold in the search process. These traditional methods have different effects and advantages. Table 2 compares Otsu’s method, Rényi entropy, Tsallis entropy, and Kapur’s entropy in image segmentation. In principle, Otsu’s method determines the threshold by maximizing the variance between classes, and the other three use different entropy concepts to find the threshold based on information theory. In terms of computational complexity, Otsu’s method is relatively low, while the other three methods are relatively high. The application scenarios are different. Otsu’s method is suitable for simple bimodal images, Rényi entropy is suitable for images with regular distribution and high accuracy requirements, Tsallis entropy is used for complex distribution images, and Kapur’s entropy performs well in texture rich images. Otsu’s method is average in terms of image detail processing ability, and others are better or very good. They are similar in that they are threshold segmentation methods based on gray information, which are designed to complete the task of image segmentation and are widely used.

In this paper, the Kapur entropy method and the Otsu method are used to realize threshold segmentation. Based on information theory, the Kapur entropy method determines the threshold by maximizing the sum of entropy of foreground and background, which is suitable for texture rich images and can effectively retain details; the Otsu method is based on the image gray characteristics and maximizes the variance between classes. It is simple to calculate and has a good segmentation effect for a bimodal histogram image. The ACOADE algorithm can enhance randomness and exploration ability, can perform well in multi-threshold image segmentation experiments, and can more effectively solve the problem of color multi-threshold image segmentation.

## 3. Image Segmentation Theory and Methods

Threshold-based image segmentation is the process of obtaining the optimal threshold through a certain method, comparing the pixels of the image, and distinguishing the target from the background. Threshold-based image segmentation methods can be divided into two categories: single-threshold segmentation and multi-threshold segmentation. Single-threshold segmentation divides the histogram into target and background based on the threshold, while multi-threshold segmentation divides the image into multiple categories through the threshold and maximizes the inter-class variance between each category. In complex images containing various objects, single-threshold methods are not as effective as multi-threshold images. Therefore, multi-threshold image segmentation methods have been widely studied [36,37]. There are many methods for multi-threshold image segmentation, and this article mainly uses the Kapur entropy method and the Otsu method to achieve multi-threshold image segmentation.

The main idea of the Kapur entropy method and the Otsu method is to divide images into multiple categories. Assume an image has *L* grayscale values. Let *n_i_* represent the number of pixels in the *i*th class of grayscale values in the image. The total number of pixels *Sum_N* = *n*_0_ + *n*_1_ + … + *N_L_* − 1. Assuming there are *K* thresholds, the grayscale of a given image can be divided into *K* + 1 categories. The threshold set can be represented by [*th*_1_, *th*_2_, *th*_3_,…, *th_K_*]. The details are as follows.

### 3.1. Kapur Entropy Method

The Kapur entropy method divides the histogram of an image into different types through multiple thresholds, calculates the sum of entropy for each type, and maximizes it [38]. The specific formula of the Kapur entropy method is as follows:

(1) $$H\left(th_{1},th_{2},\ldots ,th_{K}\right)=H_{0}+H_{1}+\ldots +H_{K}$$

where

(2) $$\begin{array}{c} \begin{array}{c} H_{0}=-\sum_{i=0}^{th_{1}-1}\frac{p_{i}}{\omega_{0}}\ln\frac{p_{i}}{\omega_{0}},\;\omega_{0}=\sum_{i=0}^{th_{1}-1}p_{i} \end{array}\\ H_{1}=-\sum_{i=th_{1}}^{th_{2}-1}\frac{p_{i}}{\omega_{1}}\ln\frac{p_{i}}{\omega_{1}},\;\omega_{1}=\sum_{i=t_{1}}^{th_{2}-1}p_{i}\\ H_{2}=-\sum_{i=th_{2}}^{th_{3}-1}\frac{p_{i}}{\omega_{2}}\ln\frac{p_{i}}{\omega_{2}},\;\omega_{2}=\sum_{i=t_{2}}^{th_{3}-1}p_{i}\\ \ldots \\ H_{K}=-\sum_{i=th_{K}}^{L-1}\frac{p_{i}}{\omega_{k}}\ln\frac{p_{i}}{\omega_{k}},\;\omega_{K}=\sum_{i=th_{K}}^{L-1}p_{i} \end{array}$$

In the formula, *H*_0_, *H*_1_, ⋯, *H_k_* represent the entropy of different classes. *ω*_0_, *ω*_1_, *ω*_2_, ⋯, *ω_K_* represents the sum of probabilities for each part. And *p_i_* represents the probability of the *i*th grayscale level. The formula is as follows:

(3) $$p_{i}=\frac{n_{i}}{Sum\_N},0\leq i\leq (L-1)$$

To obtain the optimal threshold, it is necessary to maximize the objective function (4) by controlling the parameters {*th*_1_, *th*_2_ … *th_K_*}.

(4) $$f_{Kapur}(th_{1},th_{2},\ldots ,th_{K})=\arg\max\{H(th_{1},th_{2},\ldots ,th_{K})\}$$

### 3.2. Otsu Method

The main idea of the Otsu method is to divide the histogram of an image into different types through multiple thresholds, calculate the inter-class variance of each type, and sum it up. The Otsu method states that when the sum of inter-class variances is maximized, selecting the segmentation threshold at this time results in the best image segmentation effect [39,40]. The specific formula for the Otsu method is as follows:

(5) $$\sigma^{2}\left(th_{1},th_{2},\ldots ,th_{k}\right)=\sigma_{0}^{2}+\sigma_{1}^{2}+\ldots +\sigma_{k}^{2}$$

where

(6) $$\begin{array}{c} \sigma_{0}^{2}=\omega_{0}{\left(\mu_{0}-\mu_{T}\right)}^{2},\omega_{0}=\sum_{i=0}^{th_{1}-1}p_{i},\mu_{0}=\sum_{i=0}^{th_{1}-1}\frac{ip_{i}}{\omega_{0}}\\ \sigma_{1}^{2}=\omega_{1}{\left(\mu_{1}-\mu_{T}\right)}^{2},\omega_{1}=\sum_{i=th_{1}}^{th_{2}-1}p_{i},\mu_{1}=\sum_{i=th_{1}}^{th_{2}-1}\frac{ip_{i}}{\omega_{1}}\\ \sigma_{2}^{2}=\omega_{2}{\left(\mu_{2}-\mu_{T}\right)}^{2},\omega_{2}=\sum_{i=th_{2}}^{th_{3}-1}p_{i},\mu_{2}=\sum_{i=th_{2}}^{th_{3}-1}\frac{ip_{i}}{\omega_{2}}\\ \ldots \\ \sigma_{k}^{2}=\omega_{k}{\left(\mu_{k}-\mu_{T}\right)}^{2},\omega_{k}=\sum_{i=th_{k}}^{L-1}p_{i},\mu_{k}=\sum_{i=th_{k}}^{L-1}\frac{ip_{i}}{\omega_{k}} \end{array}$$

In the formula, *μ_T_* represents the average grayscale level of the image as shown below:

(7) $$u_{T}=\omega_{0}\mu_{0}+\omega_{1}\mu_{1}+\ldots +\omega_{k}\mu_{k}$$

To obtain the optimal threshold, it is necessary to maximize the objective function (8) by controlling the parameters {*th*_1_, *th*_2_ … *th_K_*}:

(8) $$f_{Otsu}(th_{1},th_{2},\ldots ,th_{K})=\arg\max\{\sigma^{2}(th_{1},th_{2},\ldots ,th_{K})\}$$

From the above formula, it can be seen that as the threshold *K* increases, the complexity increases, and the requirements for parameters become more precise. It is particularly important to find a better threshold. Therefore, this article proposes the ACOADE algorithm to search for more precise threshold parameters.

## 4. Crayfish Optimization Algorithm (COA)

### 4.1. Initialization Phase

In the initialization phase, each crayfish represents a 1 × *dim* matrix, and each column matrix represents a solution to a problem. The initialization of the COA involves randomly generating *N* sets of candidate solutions *X* between upper and lower bounds. COA initialization is as follows:

(9) $$X=[X_{1},X_{2},\ldots ,X_{N}]=\left[\begin{array}{ccccc} X_{1,1} & \ldots & X_{1,j} & \ldots & X_{1,dim}\\ \ldots & & \ldots & & \ldots \\ X_{i,1} & \ldots & X_{i,j} & \ldots & X_{i,dim}\\ \ldots & & \ldots & & \ldots \\ X_{N,1} & \ldots & X_{N,j} & \ldots & X_{N,dim} \end{array}\right]$$

Among them, *N* is the population size. *dim* is the population dimension. *X_i,j_* is the position of the *i*th crayfish in the *j* dimension. And *X_i,j_* is obtained by the following formula:

(10) $$X_{i}=lb+(ub-lb)\times rand$$

where *lb* represents the lower bound, *ub* represents the upper bound, and *rand* is a random number of [0, 1].

### 4.2. Defining Temperature and Foraging Volume

The suitable foraging temperature for crayfish is 20–30 °C, with 25 °C being the optimal foraging temperature [41]. When the temperature is above 30 °C, crayfish will choose caves to escape the heat [42]. The temperature is defined as Formula (11). The foraging amount of crayfish is approximately a normal distribution, obtained using Formula (12):

(11) temp=rand×15+20

(12) $$p=C_{1}\times (\frac{1}{\sqrt{2\times \pi }\times \sigma )}\times \exp(-\frac{{\left(temp-\mu \right)}^{2}}{2\sigma^{2}})$$

Among them, temp represents the temperature of the environment, and *µ* refers to the temperature most suitable for crayfish, *σ*. The feeding amount of crayfish at different temperatures is controlled with *C*_1_.

### 4.3. Summer Resort Stage

When *temp* > 30, crayfish will be close to the cave for summer escape. The cave is defined as follows:

(13) $$X_{shade}=(X_{G}+X_{L})/2$$

where *X_G_* represents the optimal position obtained from the population, and *X_L_* represents the optimal position obtained after the update of the previous generation population.

When *rand* < 0.5, the COA enters the summer resort stage. At this time, the crayfish will directly approach the cave, as follows:

(14) $$X_{i,j}=X_{i,j}+C_{2}\times rand\times (X_{shade}-X_{i,j})$$

(15) $$C_{2}=2-(t/maxFES)$$

where *t* is the current number of evaluations and *maxFES* is the maximum number of evaluations.

### 4.4. Competition Stage

When *temp* > 30 and *rand* ≥ 0.5, the COA enters the competitive stage. At this time, other crayfish will compete for the same cave. The formula is as follows:

(16) $$X_{i,j}=X_{i,j}-X_{z,j}+X_{shade}$$

(17) z=round(rand×(N−1))+1

### 4.5. Foraging Stage

When the temperature is between 20 and 30 degrees, the COA enters the foraging stage. The foraging stage is divided into two types. When the food is large, crayfish will tear up the food before eating. When the food is suitable for eating, crayfish will eat directly. The definition of food and the definition of food size are as follows:

(18) $$X_{food}=X_{G}$$

(19) $$Q=C_{3}\times rand\times (fitness_{i}/fitness_{food})$$

where *C*_3_ is the food factor and the value is the constant 3. *fitness_i_* represents the fitness value of the *i*th crayfish, and *fitness_food_* represents the fitness value of the food location.

When *Q* > (*C*_3_ + 1)/2, the COA shreds the food using Formula (20). Afterward, the feeding behavior of crayfish is simulated using Formula (21):

(20) $$X_{food}=\exp(-\frac{1}{Q})\times X_{food}$$

(21) $$X_{i,j}=X_{i,j}+X_{food}\times p\times (\cos(2\times \pi \times rand)-\sin(2\times \pi \times rand))$$

When *Q* ≤ (*C*_3_ + 1)/2, the crayfish move directly toward the food and eat through Formula (22):

(22) $$X_{i,j}=(X_{i,j}-X_{food})\times p+p\times rand\times X_{i,j}$$

The pseudo code of the COA is shown in Algorithm 1.
**Algorithm 1.** Crayfish Optimization Algorithm Pseudo-CodeDefine parameters: maximum number of evaluations *maxFES*, population *N*, and dimension *dim*Initialize population *X* and obtain *X_G_*, *X_L_***While** *t* < *maxFES* Defining temperature *temp* by Formula (11) **For** *i* = 1:*N*  **If** *temp* > 30   Define cave *X_shade_* according to Formula (13)   **If** *rand* < 0.5    Crayfish conducts the summer resort stage according to Formula (14)   **Else**    Crayfish compete for caves through Formula (16)   **End**  **Else**   The food intake *p* and food size *Q* are obtained by Formulas (12) and (19)   **If** *Q* > (*C*_3_ + 1)/2    Crayfish shreds food by Formula (20)    Crayfish foraging according to Formula (21)   **Else**    Crayfish foraging according to Formula (22)   **End**  **End**  Update fitness values, *X_G_*_,_ *X_L_*  *t* = *t* + 1 **End****End**

## 5. Improvement and Implementation of the COA

### 5.1. Adaptive Feed Seeking Adjustment Strategy (AFSAS)

**Figure 1:**
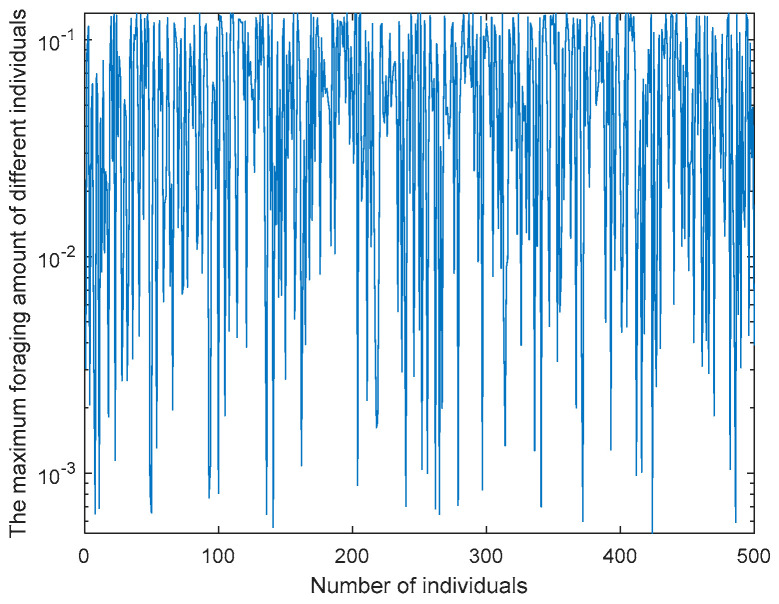
Feeding capacity curve.

In the COA, *C*_1_ is used to control the maximum foraging amount of the crayfish algorithm and plays an important role in optimization ability. This article enhances the randomness of the COA through the AFSAS. The AFSAS obtains *Fit* by the difference between its own fitness value (*fitness_i_*) and the optimal fitness value (*fitness_best_*) and then divides *Fit* + 1 by *fitness_i_* + 1 to obtain foraging quantity. Specifically, as shown in Formula (23), through the AFSAS strategy, each individual can adjust their foraging amount based on their position at the optimal position, as shown in Figure 1. By adjusting the foraging amount, each individual can adjust according to their own position, enhancing the algorithm’s exploration ability and enabling better convergence.

(23) $$p=(fitness_{i}-fitness_{best}+1)/(fitness_{i}+1)\times (\frac{1}{\sqrt{2\times \pi }\times \sigma )}\times \exp(-\frac{{\left(temp-\mu \right)}^{2}}{2\sigma^{2}})$$

### 5.2. Differential Evolution Strategy

**Figure 2:**
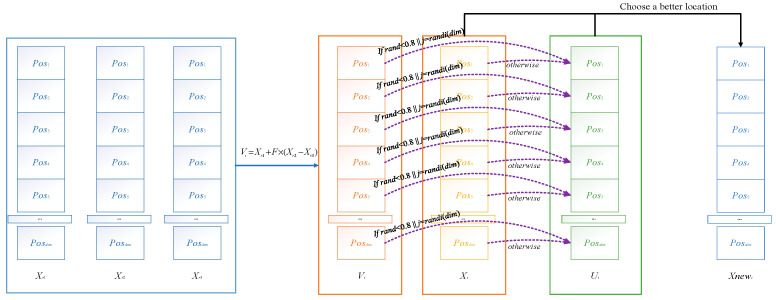
Schematic diagram of differential evolution strategy.

The differential evolution strategy improves the optimization effect of ACOADE by interacting with individuals in the population and enhancing the connections between populations through the differential evolution strategy. This can enable ACOADE to converge better and find more optimal solutions. The schematic diagram of the differential evolution strategy is shown in Figure 2. The specific formula is as follows.

#### 5.2.1. Mutation Stage

In the mutation stage, three vectors in the population are selected, and mutation is achieved through a differential operation:

(24) $$V_{i}=X_{r1}+F\times (X_{r2}-X_{r3})$$

Among them, *X_r_*_1_, *X_r_*_2_, and *X_r_*_3_ are three random individuals in the population. *F* is the variation factor with a constant value of 0.85. *V_i_* is a new individual obtained during the mutation stage.

#### 5.2.2. Crossover Stage

In the crossover stage, each individual undergoes crossover with the individuals generated by the mutation. This is selected based on probability, and each individual chooses whether to mutate according to a certain probability, as shown in Formula (25):

(25) $$U_{i,j}=\left\{\begin{array}{ll} V_{i,j},\; & if\;rand<0.8||j=randi(dim)\\ X_{i,j},\; & otherwise \end{array}\right.$$

where *U*_*i*,*j*_ represents the individuals obtained during the selection stage.

#### 5.2.3. Selection Stage

In the selection stage, greedy algorithms are used to obtain better individuals based on fitness values as the next generation, as shown in Formula (26):

(26) $$X_{i}=\left\{\begin{array}{ll} U_{i},\; & if\;f(U_{i})<f(X_{i})\\ X_{i},\; & otherwise \end{array}\right.$$

where *f*(*U_i_*) represents the fitness value obtained by the *U_i_* individual, and *f*(*X_i_*) represents the fitness value obtained by the *X_i_* individual.

### 5.3. ACOADE Pseudocode and Flowchart

**Figure 3:**
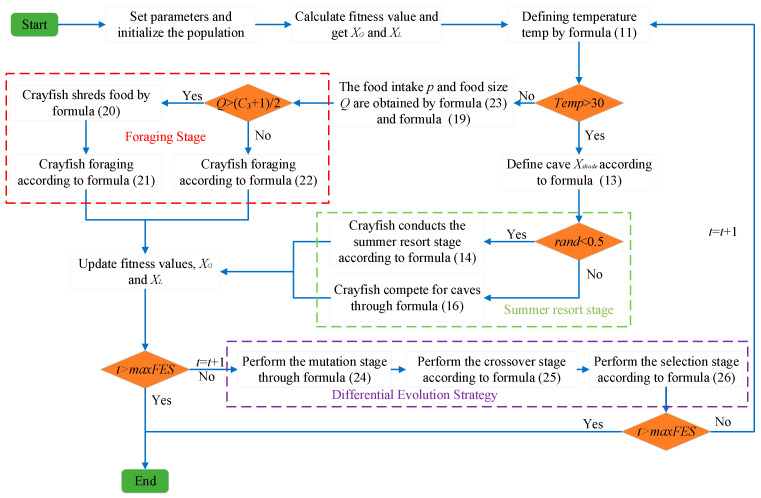
ACOADE flowchart.

The pseudocode of ACOADE is shown in Algorithm 2. The flowchart is shown in Figure 3.
**Algorithm 2.** ACOADE Pseudo-CodeDefine parameters: maximum number of evaluations *maxFES*, population *N*, and dimension *dim*Initialize population *X* and obtain *X_G_*, *X_L_***While** *t* < *maxFES* Defining temperature *temp* by Formula (11) **For** *i* = 1:*N*  **If** *temp* > 30   Define cave *X_shade_* according to Formula (13)   **If** *rand* < 0.5    Crayfish conducts the summer resort stage according to Formula (14)   **Else**    Crayfish compete for caves through Formula (16)   **End**  **Else**   The food intake *p* and food size *Q* are obtained by Formulas (19) and (23)   **If** *Q* > (*C*_3_ + 1)/2    Crayfish shreds food by Formula (20)    Crayfish foraging according to Formula (21)   **Else**    Crayfish foraging according to Formula (22)   **End**  **End**  Update fitness values, *X_G_*_,_ *X_L_*  *t* = *t* + 1 **End** **For**
*i* = 1:*N*  Perform the mutation stage through Formula (24)  Perform the crossover stage according to Formula (25)  Perform the selection stage according to Formula (26)  *t* = *t* + 1 **End****End**

## 6. Experimental Results and Discussion

This section uses the IEEE CEC2020 test function to verify the optimization effect of the ACOADE algorithm. The crayfish optimization algorithm (COA), differential evolution (DE) [43], particle swarm optimization (PSO) [44], the arithmetic optimization algorithm (AOA) [45], the whale optimization algorithm (WOA) [46], the sand cat swarm optimization (SCSO) [47], the grey wolf optimization (GWO) [48], and the moss growth optimization (MGO) [49] were selected as comparison algorithms. The ACOADE algorithm and eight comparison algorithms all had a population size of *N* = 20, dimension *dim* = 30/50, and a maximum evaluation frequency of *maxFES* = *dim* × 10,000. The comparative experimental results showed that the ACOADE algorithm had better optimization performance. The parameter settings for these algorithms are shown in Table 3.

All the experiments in this paper were completed on a computer with the 11th Gen Intel(R) Core(TM) i7-11700 processor with the primary frequency of 2.50 GHz, 16 GB memory, and the operating system of 64-bit windows 11 using matlab2021a.

### 6.1. IEEE CEC2020 Test Function Experimental Results

**Figure 4:**
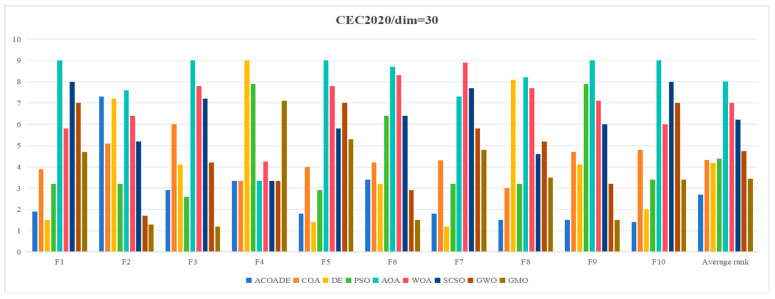
Friedman ranking of each algorithm in IEEE CEC2020 (*dim* = 30).

Table 4 shows the statistical results obtained by running ACOADE and eight comparison algorithms independently for 30 times at *dim* = 30. “Best” represents the best fitness value, “mean” represents the average fitness value, “std” represents the variance of fitness values, and “rank” represents the Friedman ranking result. From F1, it could be concluded that ACOADE could achieve better fitness values, except for the best, which did not achieve the best results. Compared with other comparative algorithms, ACOADE had better optimization performance. DE could achieve the best, indicating that DE had a good convergence effect, but its stability was insufficient. In F2, the performance of ACOADE was poor, and the results obtained were not as good as other comparison algorithms, resulting in poor optimization effects. In F2 and F3, MGO had good performance, ranking first and exhibiting good optimization effects. ACOADE also had good performance in F3, ranking slightly higher than PSO and MGO. In F4, most algorithms had good optimization effects and could achieve good fitness values. In F5, ACOADE achieved good results, except for “best”, which did not achieve the best results. Compared with other comparative algorithms, ACOADE had better optimization performance. In F6, ACOADE achieved the best, but MGO had better optimization results. In F7, DE had a better optimization effect and better performance. The optimization effect of ACOADE was relatively stable. In F8, ACOADE performed better than other compared algorithms and could achieve the best optimization results. PSO could also achieve better “best” results and have good optimization effects. In F9, ACOADE could achieve better results. The overall optimization effect of MGO was better than other comparative algorithms, with better results. In F10, ACOADE performed better and could achieve better optimization results. Figure 4 shows the ranking results of each algorithm. Compared with other algorithms, ACOADE ranked better and ultimately ranked better.

**Figure 5:**
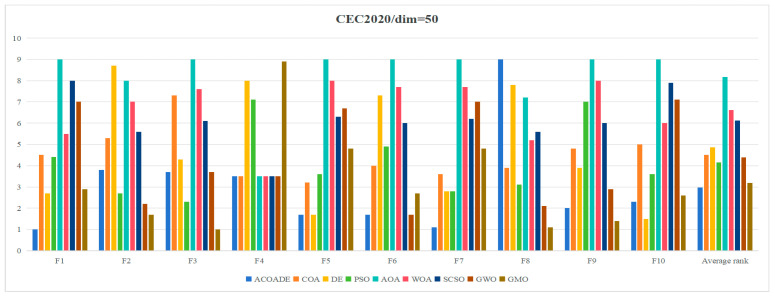
Friedman ranking of each algorithm in IEEE CEC2020 (*dim* = 50).

Table 5 shows the statistical results obtained by running ACOADE and eight comparison algorithms independently for 30 times at *dim* = 50. “Best” represents the best fitness value, “mean” represents the average fitness value, “std” represents the variance of fitness values, and “rank” represents the Friedman ranking result. In F1, ACOADE had a good optimization effect and could achieve good optimization results, only slightly higher than MGO at best. In F2, F3, F8, and F9, MGO had a good optimization effect, and the overall effect was due to other algorithms. ACOADE also had good performance in these functions, with good optimization effects. In F4, most algorithms had good performance and could find a good fitness value. In F5, the optimization effect of DE was better, but its stability was not as good as ACOADE, and the Friedman ranking was the same. In F6, ACOADE and GWO had the same ranking, and both had good performance. ACOADE had better performance in best and mean, but its stability was limited. In F7, ACOADE had a better overall optimization effect than other compared algorithms and could achieve better rankings. In F10, ACOADE ranked second and also had good optimization effects. The overall optimization effect of DE was better. Figure 5 shows the ranking results of each algorithm. Compared with other algorithms, ACOADE ranked better and ultimately ranked better. MGO also had good optimization effects, with ACOADE being superior to MGO.

### 6.2. Convergence Curve Analysis of IEEE CEC2020 Test Function

**Figure 6:**
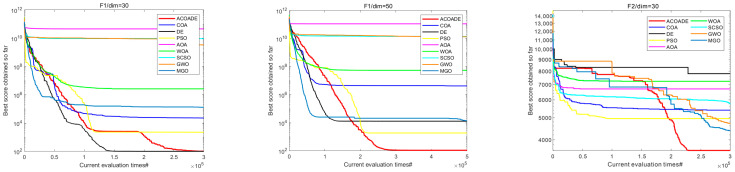

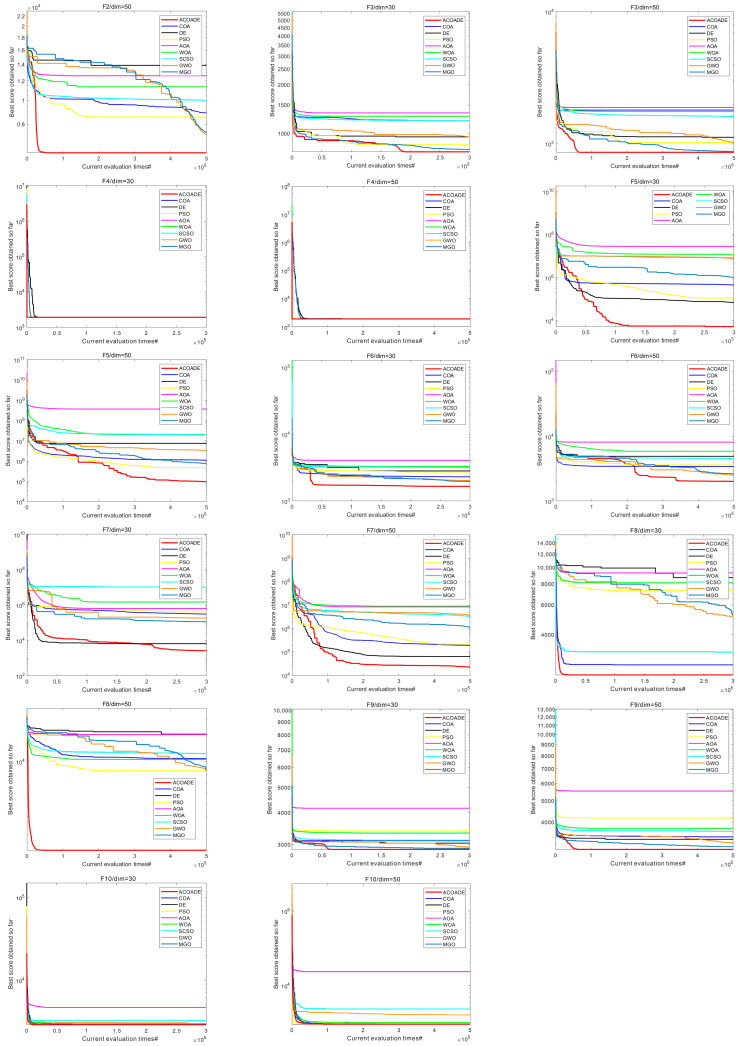
Convergence curves of each algorithm in IEEE CEC2020.

Figure 6 shows the convergence curves of eight algorithms in IEEE CEC2020, with dimensions *dim* = 30/50. In F1, the ACOADE algorithm had a good convergence curve at *dim* = 30 and could continuously converge close to the theoretical optimal solution. The ACOADE algorithm had a significantly better convergence curve than other algorithms at *dim* = 50 and could quickly converge to obtain a solution close to the theoretical optimal solution. The convergence curve obtained by the DE algorithm was also very excellent. In F2, ACOADE could quickly converge to obtain a better fitness value. GWO and MGO could continuously converge in the iteration to obtain better fitness value, but the convergence speed was slow. In F3, ACOADE converged well and could jump out of the local optimum on the way of convergence. PSO and MGO also had this good effect and could continuously converge, but the effect was not as good as MGO. Function F4 was relatively simple, and all nine algorithms had excellent convergence ability, which could quickly converge and find excellent solutions. In F5, the ACOADE algorithm obtained the best convergence curve at *dim* = 30, followed by the DE algorithm. The curves obtained by ACOADE and DE algorithms at *dim* = 50 were similar, and both could continuously converge and obtain better solutions. In F6, ACOADE could jump out of the local optimum in the process of convergence and obtain a better fitness value. The convergence of MGO and GWO was better, and better fitness values could be obtained. In F7, ACOADE, DE, and PSO algorithms all had excellent convergence performance, continuously converging at *dim* = 30 and *dim* = 50. In F8, ACOADE and the COA had a good effect at *dim* = 30 and could find very excellent solutions in the early stage. At *dim* = 50, ACOADE outperformed other comparative algorithms. In F9, the ACOADE algorithm could jump out of local optima and find better solutions. MGO could also continuously converge and obtain better fitness values. In F10, the convergence curves of the eight algorithms had a small difference and could all obtain good solutions.

### 6.3. Time Complexity Analysis

In order to verify the time complexity of each algorithm, this paper analyzes each algorithm with reference to the time complexity evaluation standard of IEEE CEC 2020 special meeting [50]. The specific steps are as follows:

(a) Calculate the system runtime *T*_0_, run the test program below:

(27) $$\begin{array}{l} x=0.05\\ \mathrm{for}\;i=1:100,000\\ x=x+x;\;x=\frac{x}{2};\;x=x*x;\;x=sqrt(x);\;x=log(x);\;x=exp(x);\;x=\frac{x}{x+2}\\ end \end{array}$$

(b) Calculate the complete computing time with 100,000 evaluations of the same dim dimensional function, which is *T*_1_.

(c) Calculate the complete computing time for the algorithm with 100,000 evaluations of the same *D* dimensional function, which is *T*_2_.

(d) The complexity of the algorithm is reflected by (*T*_2_ − *T*_1_)/*T*_0_.

Table 6 shows the (*T*_2_ − *T*_1_)/*T*_0_ results obtained by eight algorithms in the IEEE CEC2020 test function, with dimension *dim* = 30. From the table, it can be concluded that the SCSO algorithm was relatively large. The time complexity of ACOADE, the COA, and DE algorithms was similar. PSO, AOA, and GWO algorithms had lower time complexity.

## 7. Multi-Threshold Image Segmentation Experiment

**Figure 7:**
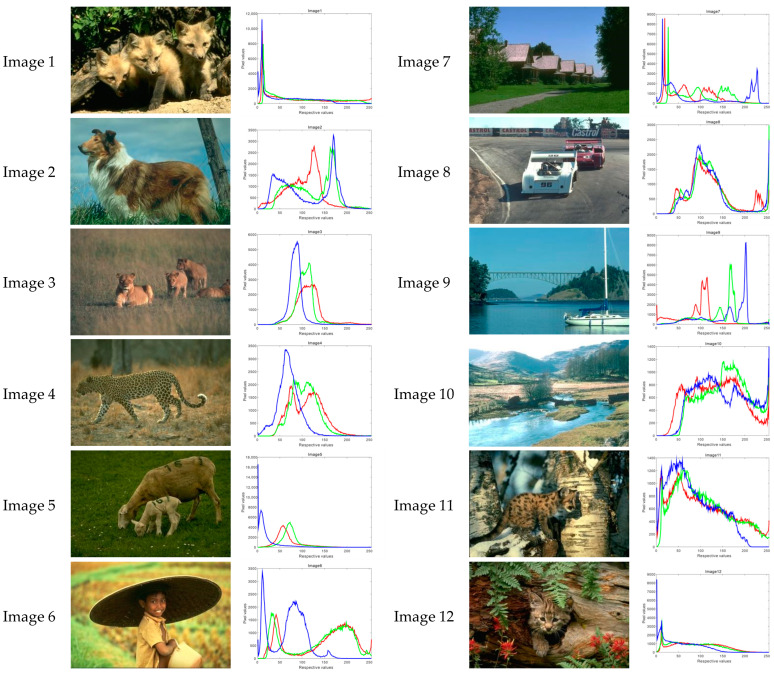
Image selection and RGB curve.

This section will use ACOADE and eight comparison algorithms for image segmentation to verify the optimization effect of ACOADE. To verify the optimization effect of ACOADE, this study selected 12 images with a size of 321 × 481 × 3 from the Berkeley segmentation dataset and the benchmark 500 (BSDS500). The Berkeley segmentation dataset and the benchmark 500 (BSDS500) can be found at the website https://www2.eecs.berkeley.edu/Research/Projects/CS/vision/bsds/ (accessed on 31 March 2025). These original images and RGB are shown in Figure 7. The red curve represents the R of the three primary colors, the green curve represents the G of the three primary colors, and the blue curve represents the B of the three primary colors. For ease of comparison, the population size of all algorithms was *N* = 20; the threshold K was the algorithm dimension dim, which was 15, 20, 25, and 30; and the evaluation frequency was *maxFES* = *dim* × 3000.

### 7.1. Statistical Results and Analysis of Image Segmentation

Table A1 presents the average fitness values derived from using the Kapur entropy method as the fitness metric and conducting 30 independent runs, while Table A2 shows the average fitness values obtained by adopting the Otsu method as the fitness value and also running independently for 30 times. The bolded values signify that among the eight algorithms, they achieved the best results. From an overall perspective, the ACOADE algorithm generally performed quite well and could obtain better results. Specifically, ACOADE secured the best average fitness values for most images, with the exceptions being in image 10 when K equaled 20 and 25. However, other algorithms also exhibited their own advantages. For instance, among image 2, image 4, and image 6, at *K* = 15, ACOADE failed to attain the best average fitness value, indicating that other algorithms outperformed it in these specific scenarios. In image 3, the COA algorithm demonstrated its strength by obtaining the best average fitness values at *K* = 15 and *K* = 20, suggesting that the COA was effective in optimizing the fitness values for image 3 under these conditions. In image 5, the GWO algorithm shone by achieving the best average fitness value at *K* = 30, showing its superiority in dealing with the fitness evaluation of image 5 at this parameter setting. Additionally, in image 8 and image 10, the COA managed to obtain the best average fitness values at *K* = 15, *K* = 20, and *K* = 25, highlighting its good performance in these two images across multiple parameter values.

Through a comprehensive analysis of the data in the two tables, it is evident that while ACOADE could achieve better fitness values in most images, demonstrating its relatively better optimization effect, other algorithms like the COA and GWO also had their moments of excellence in specific images and parameter settings, contributing to a more diverse and competitive performance landscape among the algorithms.

### 7.2. Analysis of Image Segmentation Convergence Curve

**Figure A1:**
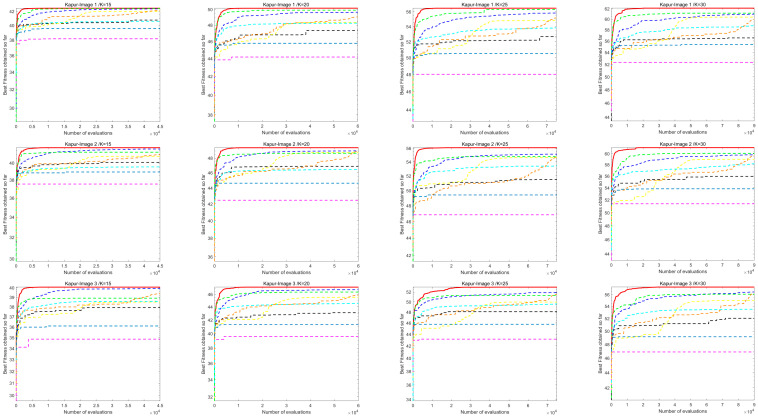

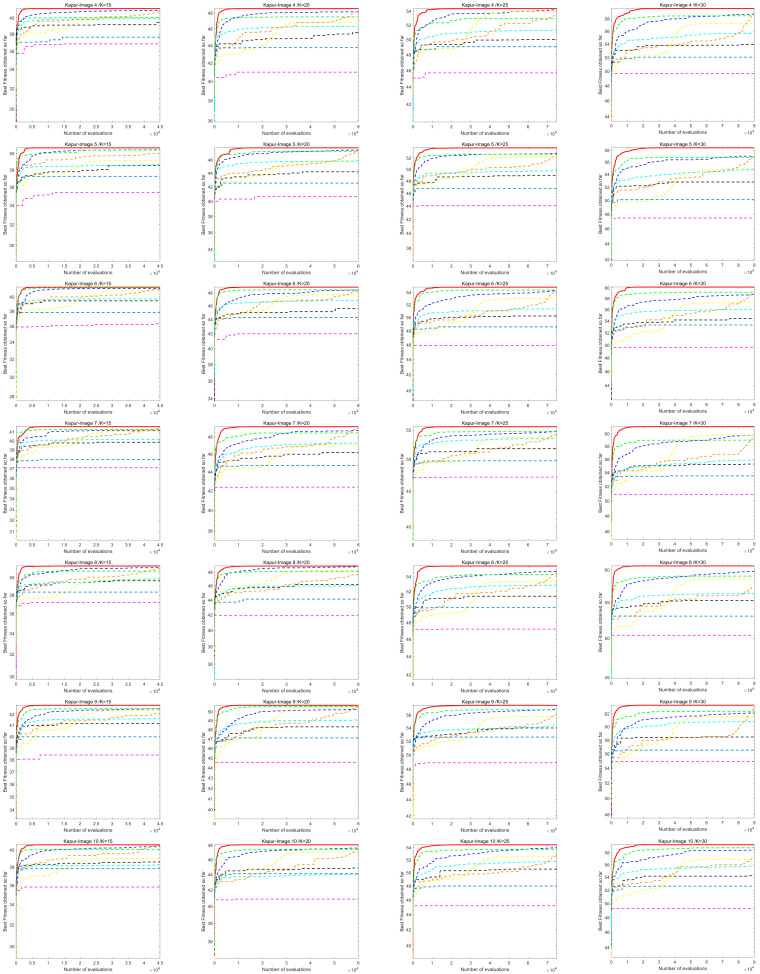

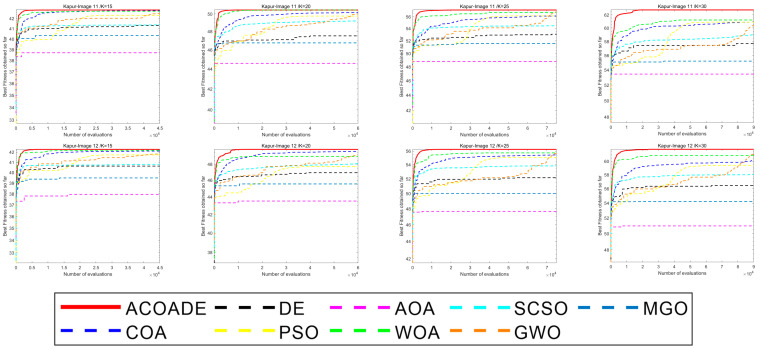
Convergence curves of each algorithm based on the Kapur entropy method.

**Figure A2:**
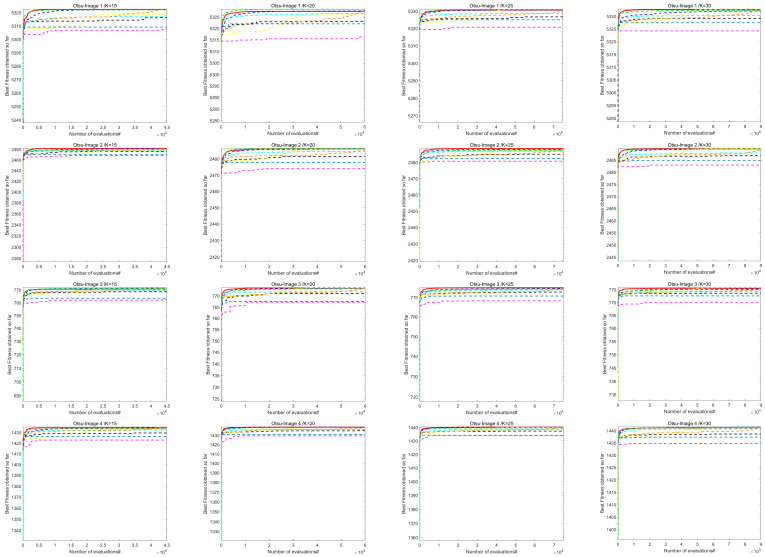

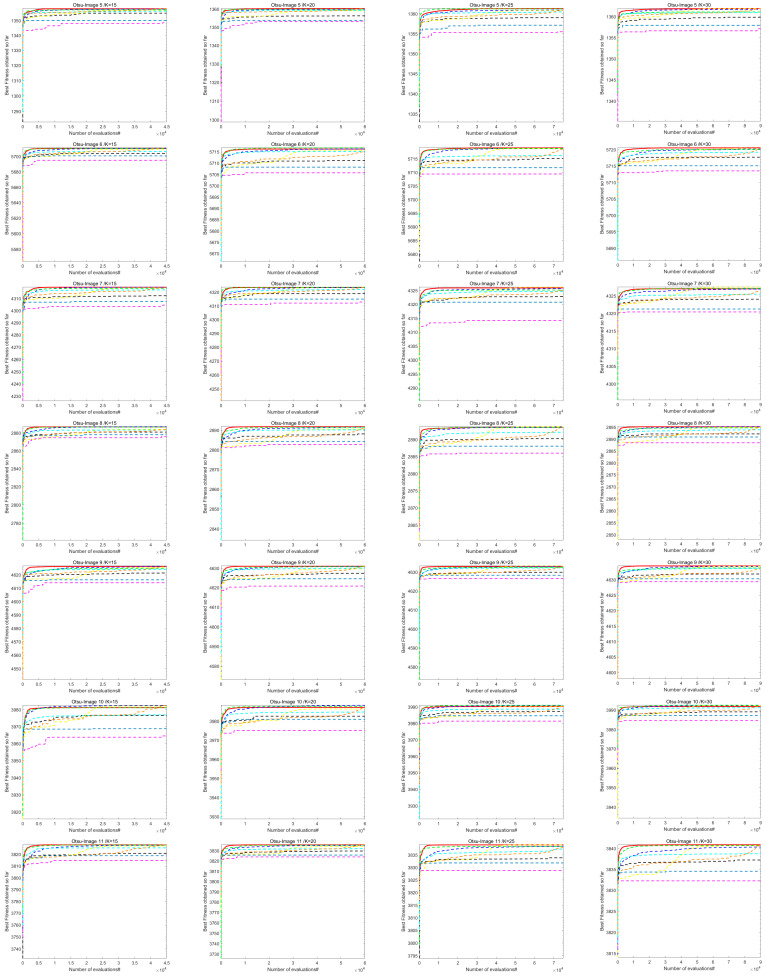

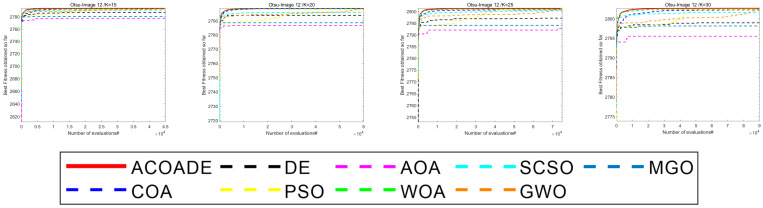
Convergence curves of each algorithm based on the Otsu method.

Figure A1 shows the convergence curve of image segmentation obtained by using the Kapur entropy method between ACOADE and eight comparison algorithms. Figure A2 shows the image segmentation convergence curves obtained by ACOADE and eight comparison algorithms using the Otsu method. It could be inferred from these two figures that among the four thresholds, ACOADE could achieve a very excellent fitness value in the early stage. The COA, WOA, GWO, and PSO algorithms also had good convergence performance. But overall, it was not as good as ACOADE. The DE, AOA, and SCSO algorithms were difficult to converge, resulting in insufficient fitness values. Overall, it can be seen that ACOADE had better optimization effects.

### 7.3. Analysis of ACOADE Image Segmentation Results

**Figure A3:**
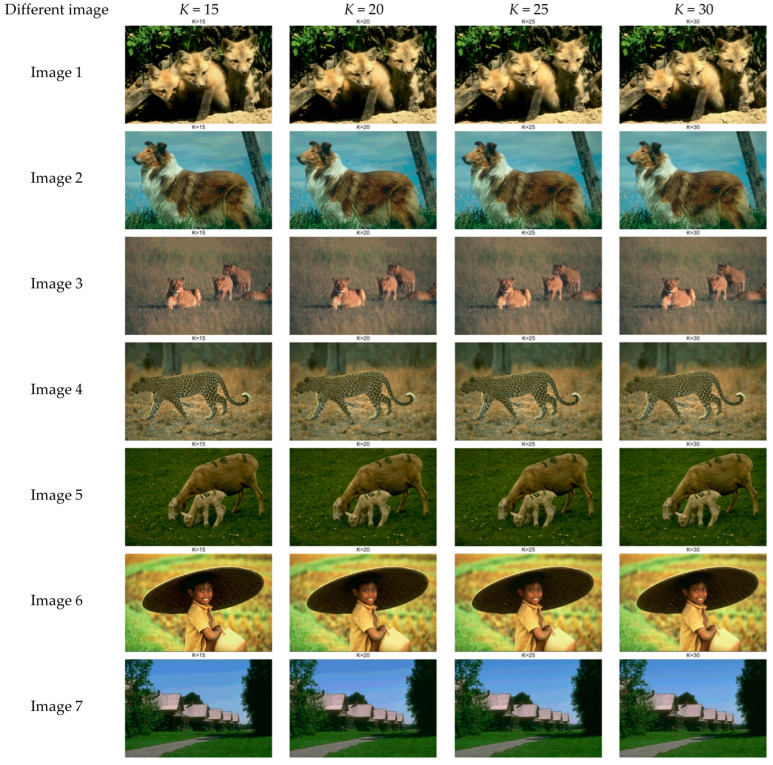

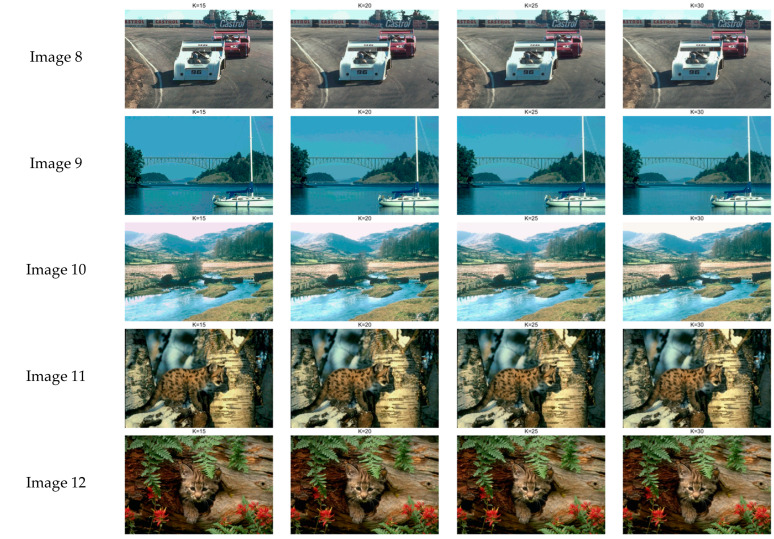
ACOADE segmentation results based on the Kapur entropy method.

**Figure A4:**
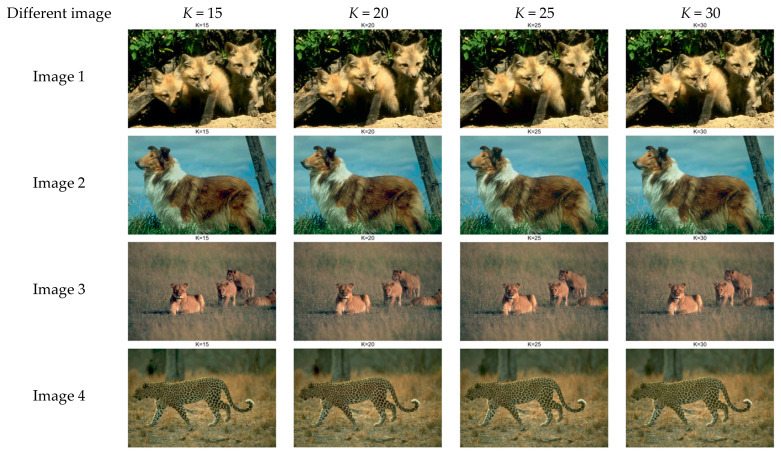

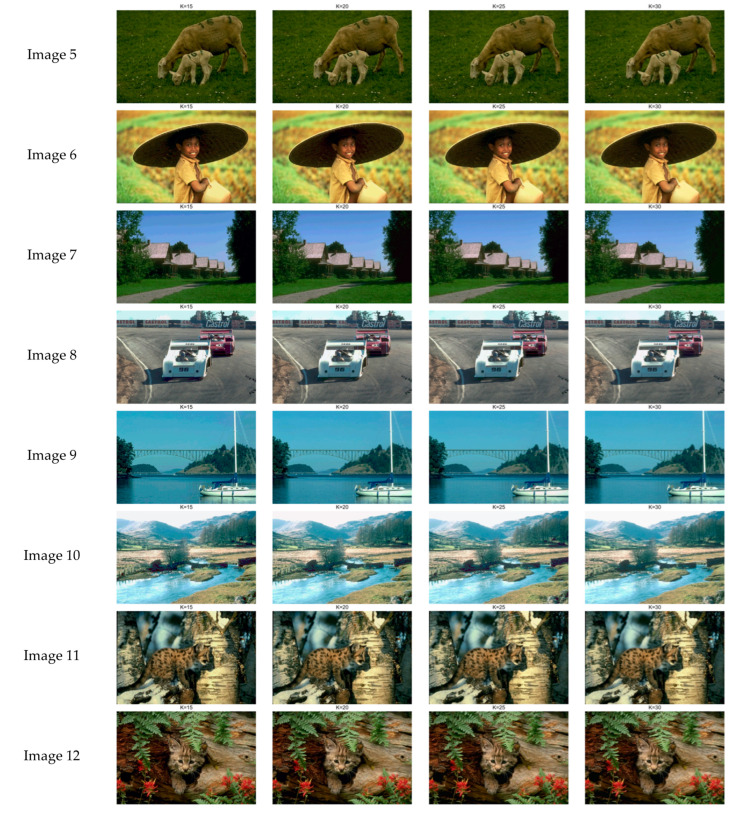
ACOADE segmentation results based on the Otsu method.

Figure A3 shows the image segmentation results obtained by ACOADE using the Kapur entropy method. Figure A4 is an image segmentation result obtained by ACOADE using the Otsu method. It can be seen that the result of ACOADE segmentation was better than that of the image. With the increase in the threshold, ACOADE segmentation results were better.

### 7.4. Results and Analysis of Wilcoxon Rank Sum Test

Table 7 and Table 8 are Wilcoxon rank sum test tables obtained by using the Kapur entropy method and the Otsu method with ACOADE and eight comparison algorithms at threshold *K* = 30. The Wilcoxon rank sum test was used to detect differences between algorithms. The *p* in the table represents the Wilcoxon rank sum test results obtained, *h* = 1 indicates significant differences between algorithms, and *h* = 0 indicates no significant differences between algorithms. From the two tables, it can be concluded that there were significant differences between ACOADE and the eight comparison algorithms, with only some images showing no significant differences. Compared with the traditional COA, it had significant differences, indicating significant improvements.

### 7.5. Segmentation Effect Evaluation Index

The Kapur entropy method and the Otsu method were used to obtain the average fitness value as the index. This part used three indicators: the feature similarity index measure (FSIM), the peak signal to noise ratio (PSNR), and the structural similarity index measure (SSIM) [51,52,53]. Details are as follows.

FSIM measures the quality of the segmented image by comparing the similarity of features between the original image and the segmented image. The specific formula is as follows:

(28) $$FSIM=\frac{\sum_{x\in \Omega }S_{L}\left(x\right)\times PC_{m}\left(x\right)}{\sum_{x\in \Omega }PC_{m}\left(x\right)}$$

where Ω represents the whole image pixel domain, *S_L_*(*x*) is the similarity index, and *PC_m_*(*x*) is the phase consistency feature, which is defined as

(29) $$PC_{m}(x)=\max\left(PC_{1}(x),PC_{2}(x)\right)$$

Among them, *PC*_1_(*x*) and *PC*_2_(*x*) represent the phase consistency characteristics of the two regions.

(30) $$S_{L}\left(x\right)={\left[S_{PC}\left(x\right)\right]}^{\alpha }\cdot {\left[S_{G}\left(x\right)\right]}^{\beta }$$

(31) $$S_{PC}\left(x\right)=\frac{2PC_{1}\left(x\right)\times PC_{2}\left(x\right)+T_{1}}{PC_{1}^{2}\left(x\right)\times PC_{2}^{2}\left(x\right)+T_{1}}$$

(32) $$S_{G}\left(x\right)=\frac{2G_{1}\left(x\right)\times G_{2}\left(x\right)+T_{2}}{G_{1}^{2}\left(x\right)\times G_{2}^{2}\left(x\right)+T_{2}}$$

Among them, *S_PC_*(*x*) represents the similarity measure of phase consistency; *SG*(*x*) represents the gradient size between two regions *G*_1_(*x*) and *G*_2_(*x*); and *α*, *β*, *T*_1_, and *T*_2_ are constants.

PSNR is an indicator used to measure the similarity between segmented images and original images, which is defined as

(33) $$\begin{array}{c} PSNR=10\log_{10}\left(\frac{255^{2}}{MSE}\right)\\ MSE=\frac{1}{MN}\sum_{i=1}^{M}\sum_{j=1}^{N}{\left[I\left(i,j\right)-K\left(i,j\right)\right]}^{2} \end{array}$$

Among them, MSE represents mean square error, *I*(*i*,*j*) represents the grayscale level of the *i*th row and *j*th column of the original image, *K*(*i*,*j*) represents the grayscale level of the *i*th row and *j*th column of the segmented image, and *M* and *N* are the number of rows and columns in the image matrix.

SSIM compares the similarity between two images through brightness, contrast, and similarity. The specific formula is as follows:

(34) $$SSIM\left(x,y\right)=\frac{\left(2\mu_{x}\mu_{y}+c_{1}\right)\left(2\sigma_{xy}+c_{2}\right)}{\left(\mu_{x}^{2}+\mu_{y}^{2}+c_{1}\right)\left(\sigma_{x}^{2}+\sigma_{y}^{2}+c_{2}\right)}$$

where *x* and *y* represents two images, and *μ_x_* and *μ_y_* represent the average values of *x* and *y*. *σ_x_*^2^ and *σ_y_*^2^ represent the variance of *x* and *y*. *σ_xy_* is the covariance of *x* and *y*, and *c*_1_ and *c*_2_ are constants.

### 7.6. Statistical Results and Analysis of Each Algorithm

**Figure A5:**
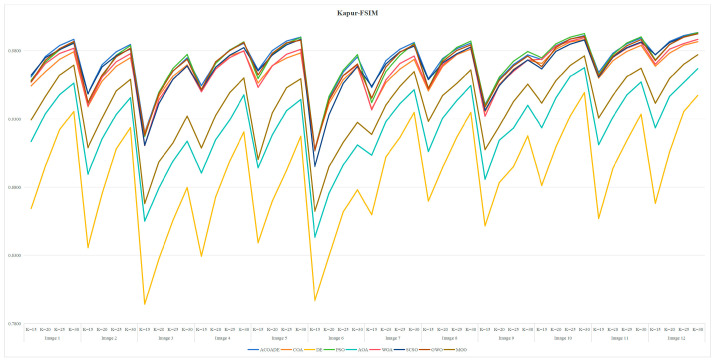
FSIM line graph based on the Kapur entropy method.

**Figure A6:**
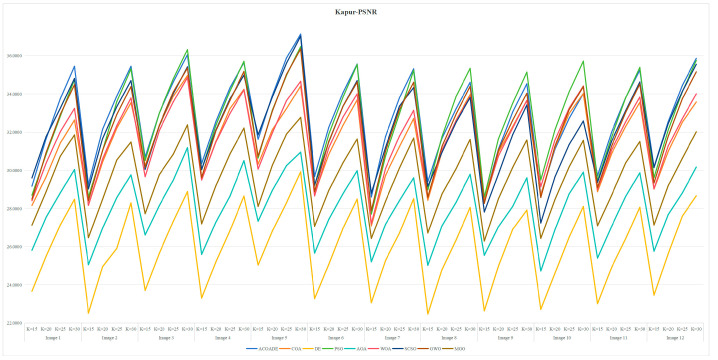
PSNR line graph based on the Kapur entropy method.

**Figure A7:**
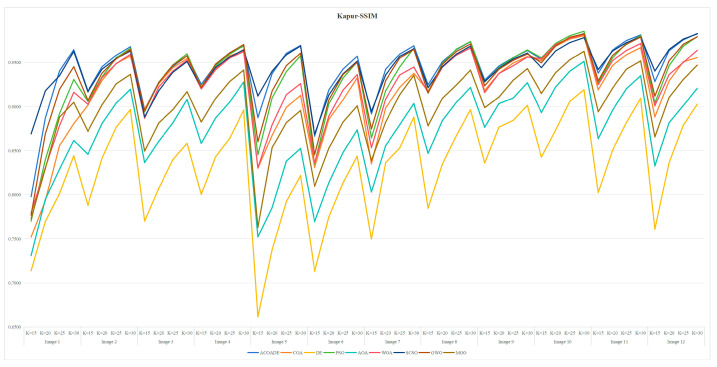
SSIM line graph based on the Kapur entropy method.

**Figure A8:**
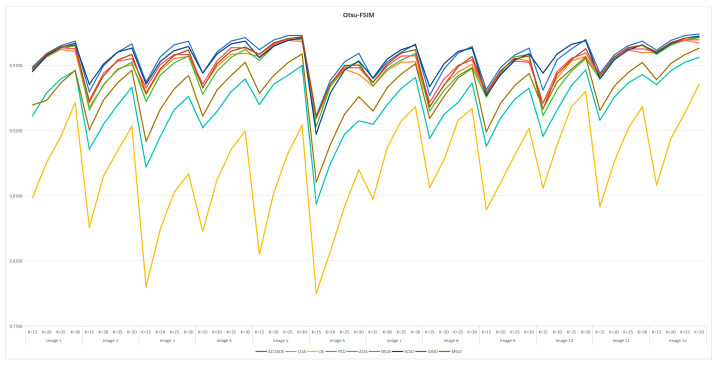
FSIM line graph based on the Otsu method.

**Figure A9:**
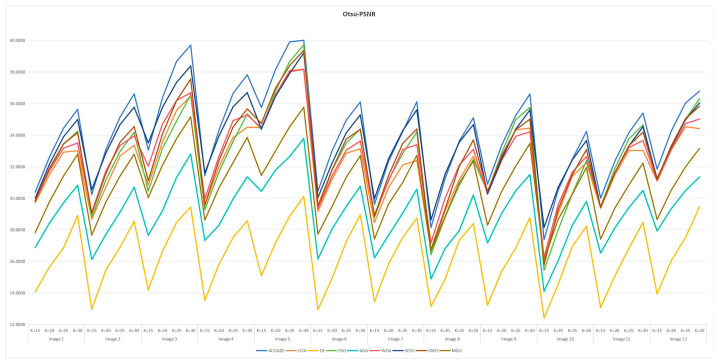
PSNR line graph based on the Otsu method.

**Figure A10:**
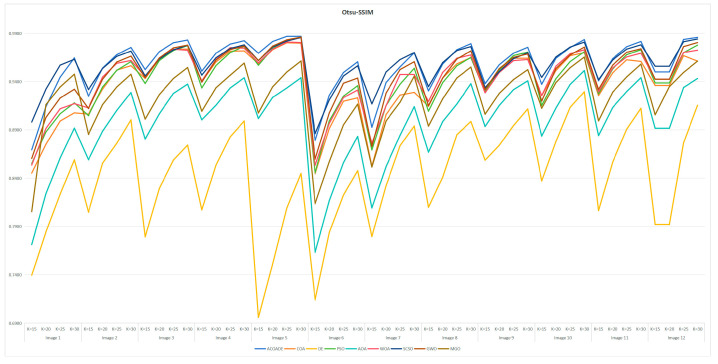
SSIM line graph based on the Otsu method.

Table A3 and Table A6 are FSIM data. Table A4 and Table A7 are PSNR data. Table A5 and Table A8 are SSIM data. These tables are based on the average values of FSIM, PSNR and SSIM obtained from the above formula by using Kapur entropy method and Otsu method to segment the image. Bold is the optimal value obtained by comparing the eight algorithms and the proportion of the optimal value in the last row. From the six tables, it can be clearly concluded that ACOADE outperformed other algorithms significantly, having the most optimal values and demonstrating the best optimization effect. Specifically, for Table A3, Table A4 and Table A5, which are based on the Kapur entropy method, ACOADE is the best. Following ACOADE, PSO took second place, showing very excellent performance. PSO had a relatively high proportion of optimal values in these tables, indicating that it could achieve good results in image segmentation using the Kapur entropy method, especially in terms of FSIM, PSNR, and SSIM values. Although not as good as ACOADE, PSO could still be a viable option when ACOADE was not applicable. For Table A6, Table A7 and Table A8, which are based on the Otsu method, ACOADE was also the best. SCSO ranked second and had a higher proportion of the optimal value compared with other algorithms except ACOADE. SCSO showed good performance in image segmentation using the Otsu method, particularly excelling in obtaining relatively high FSIM, PSNR, and SSIM values. In general, ACOADE achieved the best FSIM, PSNR, and SSIM in image segmentation based on the Kapur entropy method and the Otsu method. The FSIM, PSNR, and SSIM obtained by PSO based on the Kapur entropy method were better, but the FSIM, PSNR, and SSIM obtained by the Otsu method were poor. This indicated that PSO was more suitable for image segmentation tasks using the Kapur entropy method. The FSIM, PSNR, and SSIM obtained by SCSO based on the Otsu method were also good, but the FSIM, PSNR, and SSIM obtained by the Kapur entropy method were poor, suggesting that SCSO performed better with the Otsu method for image segmentation. Figure A5, Figure A6, Figure A7, Figure A8, Figure A9 and Figure A10 are line graphs of eight algorithms corresponding to FSIM, PSNR, and SSIM. From these figures, we can see that ACOADE had always been at the highest value, indicating that the result of ACOADE was excellent and the segmentation effect was good.

## 8. Conclusions

The complexity of multi-level threshold image segmentation increases with the increase in the threshold, and it is difficult to select a better threshold. An improved crayfish optimization algorithm was proposed. First, the parameter *p* of the COA was modified to increase the flexibility of the ACOADE algorithm. After that, the core formula of the DE algorithm was added to improve the convergence ability of the ACOADE algorithm. To prove the optimization effect of ACOADE, in the experimental part, the IEEE CEC2020 test function was selected for the experiment, and eight comparative algorithms were used for the comparative experiment. The results showed that ACOADE had a better optimization effect. In the experimental part of image segmentation, the Kapur entropy method and the Otsu method were selected to evaluate the performance of the improved algorithm. The multi-threshold performance of the algorithm was tested by 12 BSD500 images. Then, the segmentation results were analyzed according to the average values of the fitness function, FSIM, PSNR, and SSIM as indicators. The experimental results showed that ACOADE had good application prospects and could better solve the problem of multi-threshold image segmentation. In future work, we will look for better methods to improve the optimization effect of the COA and solve engineering problems.

## Figures and Tables

**Table 1:** Overview of applications in multi-level threshold image segmentation.

References	Year	Method	Criteria	Metrics
Li and Tan [31]	2019	Improved flower pollination algorithm (IFPA)	Tsallis entropy	RMSE, PSNR, SSIM, and FSIM
Yue and Zhang [32]	2020	Bat algorithm with genetic crossover and smart inertia weight (SGA-BA)	Otsu’s method and Kapur’s entropy	PSNR and SSIM
Peng and Zhang [33]	2022	Levy flight firefy algorithm (ALFA)	Rényi entropy	PSNR, SSIM, and FSIM
Gharehchopogh and Ibrikci [34]	2023	Improved African vultures optimization algorithm (AVOA)	Kapur’s entropy, Tsallis entropy, and Ostu’s entropy	PSNR, MSE, and FSIM
Jia et al. [35]	2024	Improved artificial rabbits optimization algorithm (IARO)	Otsu’s method	PSNR, FSIM, and SSIM

**Table 2:** Comparison of different methods.

Different Methods	Otsu’s Method	Rényi Entropy	Tsallis Entropy	Kapur’s Entropy
Principle	Based on the image gray histogram, the optimal segmentation threshold is determined by maximizing the inter-class variance.	From the perspective of information theory, based on the concept of Rényi entropy, the segmentation threshold is determined by optimizing the entropy correlation function.	Starting from the information theory, the Tsallis entropy formula is used to find the appropriate threshold to achieve segmentation.	Based on information theory, the segmentation threshold is determined by maximizing the entropy sum of foreground and background.
Computational complexity	Low, relatively simple calculation, mainly based on histogram statistics and simple variance calculation.	High, involving complex entropy function calculations and parameter adjustments.	The Tsallis entropy formula is relatively complex, and the calculation process requires more resources.	The entropy of foreground and background needs to be calculated, which involves many mathematical operations.
Applicable scenarios	It is applicable to images with obvious bimodal histogram and simple images with obvious contrast between target and background.	It has good effects on images with certain distribution rules and high segmentation accuracy requirements.	It has applications in processing some image scenes with complex distribution characteristics, especially non-extensive ductility.	Suitable for images with rich texture and complex gray distribution.

**Table 3:** Parameter settings for each algorithm.

Algorithm	Parameters	Value
ACOADE	*C*_3_	3
	*µ*	25
	*σ*	3
	*PCr*	0.8
	*F*	0.85
COA	*C*_1_	0.2
	*C*_3_	3
	*µ*	25
	*σ*	3
DE	*PCr*	0.8
	*F*	0.85
PSO	*Vmax*	6
	*Wmax*	0.9
	*Wmin*	0.2
	*C*_1_	2
	*C*_2_	2
AOA	*MOP_Max*	1
	*MOP_Min*	0.2
	*A*	5
	*Mu*	0.499
WOA	$\vec{A}$	1
	$\vec{C}$	[−1, 1]
	*b*	0.75
	*l*	[−1, 1]
SCSO	*SM*	2
	Roulette wheel selection	[0, 360]
	*C*	0.35
GWO	*C*_1_	[0, 2]
	*C*_2_	[0, 2]
	*C*_3_	[0, 2]
MGO	*w*	2
	*d*_1_	0.2

**Table 4:** Statistical results of each algorithm in IEEE CEC2020 (*dim* = 30).

Function	F1				F2			
Metric	Best	Mean	std	Rank	Best	Mean	std	Rank
ACOADE	100.007263	103.1353234	4.912388698	1.9	3756.681106	6774.107558	1944.159648	7.3
COA	1084.47612	219,272.7285	602,831.2621	3.9	4551.813267	5527.091371	587.9375088	5.1
DE	100	158,238,244.5	500,392,949.4	1.5	3406.578862	6382.046896	1707.389975	7.2
PSO	245.6273524	137,592,067.7	435,101,028.2	3.2	3759.52108	4525.045276	631.322931	3.2
AOA	34,631,585,306	47,162,366,374	6,341,358,236	9	5462.420262	6137.932206	538.7409906	7.6
WOA	947,040.2465	4,816,532.193	6,603,760.853	5.8	5219.901553	5835.493603	450.9568544	6.4
SCSO	1,564,528,317	6,600,643,069	4,762,509,734	8	4130.461023	5550.709956	762.8159459	5.2
GWO	191,754,180.3	2,610,624,435	1,645,105,416	7	2757.61436	3864.106404	600.1314206	1.7
MGO	15,610.26509	356,743.1976	393,486.7551	4.7	3335.405068	3808.816829	210.1428016	1.3
Function	F3				F4			
Metric	Best	Mean	std	Rank	Best	Mean	std	Rank
ACOADE	763.284243	848.9186497	67.95143827	2.9	1900	1900	0	3.35
COA	919.4692368	1091.424151	124.6918972	6	1900	1900	0	3.35
DE	754.9401571	886.1956426	67.0704855	4.1	1902.696826	1904.607056	2.190858544	9
PSO	780.7395025	830.5255228	45.89223355	2.6	1901.308916	1903.107994	1.07327413	7.9
AOA	1235.771067	1305.033272	48.31931908	9	1900	1900	0	3.35
WOA	1088.83132	1248.774694	79.19996359	7.8	1900	1900.605887	1.263985641	4.25
SCSO	1106.649395	1201.087993	72.52661575	7.2	1900	1900	0	3.35
GWO	820.2829817	884.4490052	55.25891869	4.2	1900	1900	0	3.35
MGO	777.5110871	787.4342206	10.50151862	1.2	1901.977607	1902.682872	0.809449789	7.1
Function	F5				F6			
Metric	Best	Mean	std	Rank	Best	Mean	std	Rank
ACOADE	4164.379455	23,757.71089	21,959.39943	1.8	1648.155887	2193.465238	576.5613863	3.4
COA	43,843.80452	256,730.8973	116,280.3767	4	1874.577681	2131.651082	169.7931189	4.2
DE	3576.223911	61,950.51696	150,958.5275	1.4	1682.789923	2159.661512	440.9975087	3.2
PSO	20,536.97936	95,315.30191	97,105.57137	2.9	1987.19674	2616.960139	397.2924067	6.4
AOA	3,628,809.563	19,917,260.44	21,830,638.95	9	2456.65996	3520.15197	836.5715226	8.7
WOA	1,927,916.508	4,558,598.986	1,924,015.63	7.8	2711.136829	3206.8736	413.9568654	8.3
SCSO	79,434.99067	2,554,959.911	4,720,406.392	5.8	2295.447396	2656.762958	384.9128679	6.4
GWO	232,862.8381	3,204,092.772	2,824,730.808	7	1780.466651	2023.552628	210.260063	2.9
MGO	160,472.693	451,661.6794	292,756.3129	5.3	1758.393449	1894.788584	111.4819454	1.5
Function	F7				F8			
Metric	Best	Mean	std	Rank	Best	Mean	std	Rank
ACOADE	2710.705874	5410.409589	3031.217981	1.8	2300	2300.245336	0.77582136	1.5
COA	14,464.12888	206,570.6818	289,122.7548	4.3	2300.30242	3172.771872	1829.548634	3
DE	2595.223243	5200.45548	4695.388638	1.2	4903.208318	7798.033087	1786.568797	8.1
PSO	15,858.03223	101,815.7432	231,315.2152	3.2	2300	4211.993537	2436.346963	3.2
AOA	121,566.3159	630,893.003	688,559.0364	7.3	6304.806288	8372.120381	833.0388244	8.2
WOA	371,355.3208	1,386,345.188	1,106,802.598	8.9	6232.104121	7810.044412	1066.136433	7.7
SCSO	48,470.58853	1,231,467.399	1,949,594.042	7.7	2644.138865	3608.861449	1282.526076	4.6
GWO	61,465.08637	280,347.6058	376,316.4144	5.8	2424.428004	5163.371127	1048.297659	5.2
MGO	34,210.87206	123,192.418	76,410.21241	4.8	2302.667919	3214.311711	1460.129952	3.5
Function	F9				F10			
Metric	Best	Mean	std	Rank	Best	Mean	std	Rank
ACOADE	2857.044954	2889.570165	28.1788323	1.5	2883.389856	2885.647872	2.395665355	1.4
COA	2919.871822	3019.011359	73.78378448	4.7	2883.865846	2912.342085	28.74576757	4.8
DE	2895.944673	2983.98118	47.24669962	4.1	2886.766131	2886.861177	0.084784519	2
PSO	3100.844554	3233.749179	115.7491289	7.9	2883.537132	2893.455459	16.88343948	3.4
AOA	3623.901498	3832.296184	196.6451225	9	3983.76846	4465.827328	330.842407	9
WOA	3037.304047	3184.185281	100.0622848	7.1	2912.037487	2956.441033	26.67178878	6
SCSO	2977.290919	3080.155951	77.0621037	6	2993.341647	3126.343985	107.5714625	8
GWO	2906.382048	2934.767193	29.07575942	3.2	2969.008408	3021.194491	74.53125275	7
MGO	2871.763954	2884.993922	9.671039363	1.5	2884.810923	2887.682042	1.703034423	3.4

**Table 5:** Statistical results of each algorithm in IEEE CEC2020 (*dim* = 50).

Function	F1				F2			
Metric	Best	Mean	std	Rank	Best	Mean	std	Rank
ACOADE	108.8591541	8569.383087	9675.824656	1	6770.160182	7823.680672	688.4177612	3.8
COA	52,500.58819	58,739,156.9	130,200,343.6	4.5	7573.985848	8718.429891	675.2157105	5.3
DE	955.1006988	250,369,281.7	534,847,829.4	2.7	9080.786937	13,206.21145	1581.861587	8.7
PSO	650.6615395	1,382,613,143	2,167,932,314	4.4	6259.726145	7716.786559	1013.624647	2.7
AOA	91,991,830,310	1.03125 × 10^11^	7420731514	9	11158.09512	12350.56576	688.9864391	8
WOA	2,470,535.482	14,623,181.21	12,367,899.3	5.5	8095.389705	10,655.15053	1586.56782	7
SCSO	6,779,199,246	16,850,409,460	6,659,164,304	8	7212.524363	9913.44106	1465.892689	5.6
GWO	4,419,095,763	10,575,805,557	4,736,818,416	7	5563.828612	6747.19862	747.2991989	2.2
MGO	100.1276464	10,041.64104	16,210.90192	2.9	5129.517418	6705.242262	886.4073441	1.7
Function	F3				F4			
Metric	Best	Mean	std	Rank	Best	Mean	std	Rank
ACOADE	892.2015081	1209.500868	347.7231816	3.7	1900	1900	0	3.5
COA	1416.483958	1638.896018	140.791363	7.3	1900	1900	0	3.5
DE	987.0028095	1110.385468	48.12427382	4.3	1905.375206	1907.072425	1.822346996	8
PSO	924.059378	951.9311006	25.5364916	2.3	1904.485183	1906.510349	1.093544775	7.1
AOA	1727.93147	1890.969773	65.15968959	9	1900	1900	0	3.5
WOA	1507.394946	1742.987906	111.204918	7.6	1900	1900	0	3.5
SCSO	1411.790261	1657.161138	181.4439377	6.1	1900	1900	0	3.5
GWO	1019.060483	1117.335817	74.62186788	3.7	1900	1900	0	3.5
MGO	834.6273508	870.7315147	24.73878461	1	1905.153199	1908.231742	1.706520426	8.9
Function	F5				F6			
Metric	Best	Mean	std	Rank	Best	Mean	std	Rank
ACOADE	46,265.73363	230,204.9381	148,213.0597	1.7	1931.411877	2548.212182	564.687593	1.7
COA	219,456.4503	538,188.6149	235,349.8507	3.2	2489.89069	3008.934785	488.5531674	4
DE	26,726.20628	162,352.048	260,494.3242	1.7	2689.898661	4838.539351	957.8797086	7.3
PSO	101,560.0941	680,679.0893	568,772.752	3.6	3131.761355	3390.394474	187.2322246	4.9
AOA	25,812,439.67	185,908,457.8	121,368,963.6	9	5714.070303	7490.698223	1166.250615	9
WOA	7,559,377.837	20,785,676.94	9,043,611.979	8	4243.679276	4760.77615	345.1833703	7.7
SCSO	1,306,347.499	8,552,658.318	10,276,599.82	6.3	3469.890083	3980.14333	521.9487819	6
GWO	645,009.5186	5,766,867.68	5,351,178.706	6.7	2240.089116	2562.935749	256.4970737	1.7
MGO	612,166.4773	1,202,904.444	412,948.4715	4.8	2497.863554	2734.188196	141.2120482	2.7
Function	F7				F8			
Metric	Best	Mean	std	Rank	Best	Mean	std	Rank
ACOADE	27,074.05439	95,056.32106	68,146.50439	1.1	2300	14,481.42345	4296.77277	9
COA	189,136.1957	362,776.274	117,871.6525	3.6	2324.823852	10,363.19992	2955.397892	3.9
DE	16,146.58439	611,695.5658	1,097,899.154	2.8	9762.339722	14,934.53488	1849.754285	7.8
PSO	67,952.23572	158,122.3158	86,467.75045	2.8	7800.896342	8974.526664	899.8619752	3.1
AOA	642,143.8399	7,931,255.382	8,117,460.836	9	14,359.00335	15,286.02641	569.1695023	7.2
WOA	1,301,460.272	3,386,654.2	1,548,288.03	7.7	10,593.58318	11,886.14836	1050.222312	5.2
SCSO	601,352.1146	3,087,191.852	2,414,766.625	6.2	8716.204221	10,687.28908	1061.33651	5.6
GWO	323,313.0188	3,781,651.198	3,479,083.245	7	7259.773652	8183.511859	708.851635	2.1
MGO	483,675.732	749,031.2138	181,727.2245	4.8	6755.552965	7790.437913	552.1688017	1.1
Function	F9				F10			
Metric	Best	Mean	std	Rank	Best	Mean	std	Rank
ACOADE	3034.433226	3151.715012	94.15435701	2	2960.491202	3036.621482	53.93364345	2.3
COA	3262.11068	3407.316918	90.8676576	4.8	3033.887695	3113.124504	54.69435987	5
DE	3287.581127	3302.411211	18.89958115	3.9	2960.503168	3008.508185	41.17575554	1.5
PSO	3484.518906	3710.336398	142.6309314	7	3019.270176	3080.49439	36.53769104	3.6
AOA	4288.08514	4993.154121	373.5014436	9	12,687.00492	15,290.58653	1017.554449	9
WOA	3545.013344	3685.254128	98.98664673	8	3104.505998	3158.609858	45.68493686	6
SCSO	3392.97403	3494.369315	100.8939733	6	3708.495223	4291.768992	443.1579137	7.9
GWO	3051.940275	3141.603217	42.64455885	2.9	3234.83296	3649.056696	280.5028192	7.1
MGO	3016.000828	3044.178322	19.03052342	1.4	2989.332317	3033.431009	18.42523186	2.6

**Table 6:** Comparison of time complexity of each algorithm.

Function	ACOADE	COA	DE	PSO	AOA	WOA	SCSO	GWO
F1	119.41	99.21	126.24	59.95	77.97	43.20	1474.50	111.21
F2	126.59	123.67	120.54	53.43	71.96	34.41	1458.64	106.61
F3	114.39	118.38	113.83	47.44	66.85	30.07	1461.27	104.72
F4	117.25	107.61	108.46	50.91	68.65	28.51	1453.49	102.88
F5	115.92	115.82	112.71	47.25	66.82	30.81	1458.81	104.05
F6	111.65	102.94	110.82	46.73	68.65	29.44	1454.16	105.57
F7	115.47	102.54	111.83	47.73	66.99	28.56	1456.43	104.00
F8	162.28	222.23	115.24	45.69	69.66	31.50	1457.43	105.42
F9	195.99	276.87	110.43	47.49	65.51	25.75	1457.85	103.50
F10	173.46	237.32	113.82	46.96	65.63	28.99	1460.72	104.13

**Table 7:** Wilcoxon rank sum test results based on the Kapur entropy method.

Different images		Image 1	Image 2	Image 3	Image 4	Image 5	Image 6
COA	*p*	3.52 × 10^−6^	5.31 × 10^−5^	7.73 × 10^−3^	4.20 × 10^−4^	3.18 × 10^−6^	2.16 × 10^−5^
	*h*	1	1	1	1	1	1
DE	*p*	1.73 × 10^−6^	1.73 × 10^−6^	1.73 × 10^−6^	1.73 × 10^−6^	1.73 × 10^−6^	1.73 × 10^−6^
	*h*	1	1	1	1	1	1
PSO	*p*	2.83 × 10^−4^	4.11 × 10^−3^	5.71 × 10^−4^	3.88 × 10^−4^	3.11 × 10^−5^	2.83 × 10^−4^
	*h*	1	1	1	1	1	1
AOA	*p*	1.73 × 10^−6^	1.73 × 10^−6^	1.73 × 10^−6^	1.73 × 10^−6^	1.73 × 10^−6^	1.73 × 10^−6^
	*h*	1	1	1	1	1	1
WOA	*p*	3.61 × 10^−3^	3.00 × 10^−2^	1.11 × 10^−3^	1.48 × 10^−2^	3.52 × 10^−6^	2.26 × 10^−3^
	*h*	1	1	1	1	1	1
SCSO	*p*	1.73 × 10^−6^	1.73 × 10^−6^	1.73 × 10^−6^	1.73 × 10^−6^	1.73 × 10^−6^	1.73 × 10^−6^
	*h*	1	1	1	1	1	1
GWO	*p*	5.71 × 10^−4^	3.38 × 10^−3^	**5.71 × 10^−2^**	7.27 × 10^−3^	3.68 × 10^−2^	**3.39 × 10^−1^**
	*h*	1	1	0	1	1	0
MGO	*p*	1.73 × 10^−6^	1.73 × 10^−6^	1.73 × 10^−6^	1.73 × 10^−6^	1.73 × 10^−6^	1.73 × 10^−6^
	*h*	1	1	1	1	1	1
Different images		Image 7	Image 8	Image 9	Image 10	Image 11	Image 12
COA	*p*	2.13 × 10^−6^	7.71 × 10^−4^	5.75 × 10^−6^	**9.92 × 10^−1^**	1.73 × 10^−6^	2.60 × 10^−6^
	*h*	1	1	1	0	1	1
DE	*p*	1.73 × 10^−6^	1.73 × 10^−6^	1.73 × 10^−6^	1.73 × 10^−6^	1.73 × 10^−6^	1.73 × 10^−6^
	*h*	1	1	1	1	1	1
PSO	*p*	3.50 × 10^−2^	2.22 × 10^−4^	2.07 × 10^−2^	**1.11 × 10^−1^**	6.34 × 10^−6^	2.84 × 10^−5^
	*h*	1	1	1	0	1	1
AOA	*p*	1.73 × 10^−6^	1.73 × 10^−6^	1.73 × 10^−6^	1.73 × 10^−6^	1.73 × 10^−6^	1.73 × 10^−6^
	*h*	1	1	1	1	1	1
WOA	*p*	1.36 × 10^−4^	**4.91 × 10^−1^**	6.04 × 10^−3^	2.41 × 10^−3^	7.27 × 10^−3^	2.16 × 10^−5^
	*h*	1	0	1	1	1	1
SCSO	*p*	1.73 × 10^−6^	1.73 × 10^−6^	1.73 × 10^−6^	1.92 × 10^−6^	1.73 × 10^−6^	1.73 × 10^−6^
	*h*	1	1	1	1	1	1
GWO	*p*	2.85 × 10^−2^	4.72 × 10^−2^	4.49 × 10^−2^	**3.29 × 10^−1^**	7.73 × 10^−3^	9.84 × 10^−3^
	*h*	1	1	1	0	1	1
MGO	*p*	1.73 × 10^−6^	1.73 × 10^−6^	1.73 × 10^−6^	1.73 × 10^−6^	1.73 × 10^−6^	1.73 × 10^−6^
	*h*	1	1	1	1	1	1

**Table 8:** Wilcoxon rank sum test results based on the Ostu method.

Different images		Image 1	Image 2	Image 3	Image 4	Image 5	Image 6
COA	*p*	2.13 × 10^−6^	1.80 × 10^−5^	7.51 × 10^−5^	2.5 × 10^−4^	1.02 × 10^−5^	2.16 × 10^−5^
	*h*	1	1	1	1	1	1
DE	*p*	1.73 × 10^−6^	1.73 × 10^−6^	1.73 × 10^−6^	1.73 × 10^−6^	1.73 × 10^−6^	1.73 × 10^−6^
	*h*	1	1	1	1	1	1
PSO	*p*	2.5 × 10^−4^	2.61 × 10^−4^	4.72 × 10^−2^	1.83 × 10^−3^	5.75 × 10^−6^	3.39 × 10^−1^
	*h*	1	1	1	1	1	0
AOA	*p*	1.73 × 10^−6^	1.73 × 10^−6^	1.73 × 10^−6^	1.73 × 10^−6^	1.73 × 10^−6^	1.73 × 10^−6^
	*h*	1	1	1	1	1	1
WOA	*p*	1.25 × 10^−4^	2.84 × 10^−5^	1.92 × 10^−6^	7.69 × 10^−6^	1.73 × 10^−6^	6.84 × 10^−3^
	*h*	1	1	1	1	1	1
SCSO	*p*	1.73 × 10^−6^	1.73 × 10^−6^	1.73 × 10^−6^	1.73 × 10^−6^	1.73 × 10^−6^	1.73 × 10^−6^
	*h*	1	1	1	1	1	1
GWO	*p*	2.13 × 10^−1^	3.72 × 10^−5^	5.75 × 10^−6^	3.6 × 10^−4^	4.95 × 10^−2^	3.82 × 10^−1^
	*h*	0	1	1	1	1	0
MGO	*p*	1.73 × 10^−6^	1.73 × 10^−6^	1.73 × 10^−6^	1.73 × 10^−6^	1.73 × 10^−6^	1.73 × 10^−6^
	*h*	1	1	1	1	1	1
Different images		Image 7	Image 8	Image 9	Image 10	Image 11	Image 12
COA	*p*	2.13 × 10^−6^	9.92 × 10^−1^	5.22 × 10^−6^	2.11 × 10^−3^	1.73 × 10^−6^	2.35 × 10^−6^
	*h*	1	0	1	1	1	1
DE	*p*	1.73 × 10^−6^	1.73 × 10^−6^	1.73 × 10^−6^	1.73 × 10^−6^	1.73 × 10^−6^	1.73 × 10^−6^
	*h*	1	1	1	1	1	1
PSO	*p*	1.59 × 10^−3^	1.92 × 10^−1^	2.71 × 10^−1^	4.39 × 10^−3^	1.11 × 10^−3^	4.73 × 10^−6^
	*h*	1	0	0	1	1	1
AOA	*p*	1.73 × 10^−6^	1.73 × 10^−6^	1.73 × 10^−6^	1.73 × 10^−6^	1.73 × 10^−6^	1.73 × 10^−6^
	*h*	1	1	1	1	1	1
WOA	*p*	3.52 × 10^−6^	2.83 × 10^−4^	2.13 × 10^−6^	9.27 × 10^−3^	7.51 × 10^−5^	9.32 × 10^−6^
	*h*	1	1	1	1	1	1
SCSO	*p*	1.73 × 10^−6^	1.73 × 10^−6^	1.73 × 10^−6^	1.73 × 10^−6^	1.73 × 10^−6^	1.73 × 10^−6^
	*h*	1	1	1	1	1	1
GWO	*p*	4.68 × 10^−3^	7.16 × 10^−4^	6.16 × 10^−4^	2.99 × 10^−1^	1.15 × 10^−4^	1.75 × 10^−2^
	*h*	1	1	1	0	1	1
MGO	*p*	1.73 × 10^−6^	1.73 × 10^−6^	1.73 × 10^−6^	1.73 × 10^−6^	1.73 × 10^−6^	1.73 × 10^−6^
	*h*	1	1	1	1	1	1

## Data Availability

The data presented in this study are available on request from the corresponding author.

## Appendix A

**Table A1:** Statistical results based on the Kapur entropy method.

Different Images	Metric	ACOADE	COA	DE	PSO	AOA	WOA	SCSO	GWO	MGO
Image 1	*K* = 15	42.4189	42.3872	40.9124	42.0223	38.3013	42.3564	41.3787	42.3278	40.0292
	*K* = 20	49.9905	49.8488	47.4201	49.4014	43.9298	49.8716	48.5662	49.8933	46.2150
	*K* = 25	56.4083	55.9410	52.7168	55.4794	48.6062	56.2292	54.6524	56.0221	51.3665
	*K* = 30	61.6432	60.9763	57.0001	60.9890	52.5880	61.4314	59.5284	61.0223	55.5319
Image 2	*K* = 15	41.8515	41.7944	40.3581	41.4623	37.6679	41.7211	40.6184	41.7229	39.2923
	*K* = 20	49.4134	49.2583	46.9375	48.8274	43.2134	49.2394	47.8200	49.2029	45.5202
	*K* = 25	55.6733	55.2837	51.9964	54.6056	47.6570	55.4200	53.7583	55.3189	50.5009
	*K* = 30	60.8527	60.2036	56.0620	60.1401	51.6224	60.4397	58.4480	60.3143	54.6486
Image 3	*K* = 15	39.9393	39.8987	38.0131	39.3086	35.4251	39.5718	38.4455	39.8143	36.6794
	*K* = 20	46.9729	46.8272	43.7862	46.1444	40.0022	46.3806	44.8137	46.7628	41.9860
	*K* = 25	52.5136	52.3958	48.3274	51.6439	44.0845	51.9424	49.5934	51.9108	46.5254
	*K* = 30	56.8369	56.6694	51.9759	56.0725	47.4531	56.2430	53.9496	56.3230	50.2339
Image 4	*K* = 15	41.0635	41.0121	39.4237	40.5931	36.7200	40.9068	39.8841	40.9464	38.3009
	*K* = 20	48.3645	48.2956	45.5550	47.5888	41.8119	48.2807	46.4961	48.2323	44.2028
	*K* = 25	54.3129	54.1722	50.4362	53.3772	46.4523	54.0318	52.2003	54.1124	48.9436
	*K* = 30	59.2495	58.8385	54.2841	58.3111	49.6528	58.8301	56.4138	58.8272	52.9043
Image 5	*K* = 15	40.6244	40.5649	38.7703	39.9243	36.0563	40.3940	39.6137	40.5806	37.5724
	*K* = 20	47.7630	47.4959	44.6175	46.5928	40.9916	47.2919	46.1515	47.5533	43.1110
	*K* = 25	53.5100	53.0527	49.2695	52.2626	44.9853	52.9151	51.3247	53.1891	47.5231
	*K* = 30	58.3273	57.4114	53.0085	56.9793	48.5013	57.3637	55.6195	57.6468	51.2566
Image 6	*K* = 15	41.3152	41.2257	39.4705	40.8486	36.9771	41.2391	40.0394	41.2387	38.3992
	*K* = 20	48.6627	48.5265	45.7118	48.1438	42.0967	48.4027	46.6503	48.5037	44.3872
	*K* = 25	54.6796	54.3355	50.6650	53.7134	46.4626	54.4381	52.3517	54.4732	49.0705
	*K* = 30	59.5930	58.8796	54.5860	59.0092	50.0120	59.1833	56.9444	59.1560	52.9086
Image 7	*K* = 15	41.5258	41.4462	39.9755	41.1839	37.3975	41.3574	40.4920	41.4812	38.8389
	*K* = 20	49.0401	48.8234	46.3508	48.3327	42.9216	48.6741	47.4053	48.7584	45.0599
	*K* = 25	55.3568	54.8602	51.5606	54.5709	47.6923	54.7847	53.5042	54.9380	50.2130
	*K* = 30	60.7753	59.8646	55.7715	59.8907	51.3258	59.7983	58.0460	59.9558	54.2435
Image 8	*K* = 15	41.2688	41.2551	39.7933	40.8768	37.1133	41.1455	40.1393	41.1957	38.6367
	*K* = 20	48.8240	48.7634	46.3435	48.2195	42.5521	48.6698	46.9323	48.4380	44.9944
	*K* = 25	54.9292	54.9071	51.5904	54.4518	47.2995	54.8401	52.5676	54.6813	49.7805
	*K* = 30	60.1956	59.8743	55.6199	59.6966	51.2077	59.8220	57.4685	59.4348	54.0735
Image 9	*K* = 15	42.8060	42.6672	41.3992	42.6731	38.8370	42.6231	41.8059	42.6990	40.5781
	*K* = 20	50.7075	50.5005	48.4156	50.3913	45.0766	50.4623	49.2559	50.4959	47.2360
	*K* = 25	57.3871	56.9316	54.1265	56.9199	50.2804	56.9739	55.5348	57.0800	52.8415
	*K* = 30	62.8881	62.2860	58.7238	62.7154	54.3812	62.4282	60.7157	62.6237	57.2605
Image 10	*K* = 15	40.5527	40.5203	39.1183	40.0383	36.6387	40.3660	39.0954	40.3995	38.0019
	*K* = 20	47.8911	47.9984	45.3713	47.4045	41.7556	48.0064	45.7671	47.8569	44.0868
	*K* = 25	53.8739	54.0951	50.4478	53.3803	46.3038	54.1408	51.2830	53.9038	48.9514
	*K* = 30	58.9730	58.9001	54.5390	58.6186	49.9265	58.6990	56.0074	58.5067	53.0520
Image 11	*K* = 15	42.8002	42.7519	41.3868	42.3661	39.0574	42.7678	41.8661	42.7193	40.7081
	*K* = 20	50.4782	50.2647	48.0249	49.7333	44.4983	50.3810	49.2114	50.3013	46.8861
	*K* = 25	56.9108	56.4964	53.3186	56.2184	49.3844	56.6542	55.0692	56.5306	52.0905
	*K* = 30	62.3696	61.6228	57.8068	61.6776	53.4586	61.8461	60.0776	61.8519	56.2031
Image 12	*K* = 15	42.2099	42.1479	40.7124	41.8602	38.2493	42.1147	41.3291	42.0875	39.6906
	*K* = 20	49.8190	49.5961	47.2773	49.1314	43.7807	49.6737	48.5218	49.6320	45.9661
	*K* = 25	56.1680	55.7125	52.4447	55.4466	48.5820	55.7844	54.3875	55.9603	51.1059
	*K* = 30	61.3955	60.6098	56.6211	60.5207	52.2275	60.8735	59.0753	60.9206	55.1672

**Table A2:** Statistical results based on the Otsu method.

Different Images	Metric	ACOADE	COA	DE	PSO	AOA	WOA	SCSO	GWO	MGO
Image 1	*K* = 15	5322.36	5321.97	5315.51	5320.53	5305.57	5321.98	5319.06	5321.93	5310.00
	*K* = 20	5328.06	5327.73	5322.91	5326.85	5315.31	5327.79	5326.05	5327.79	5319.59
	*K* = 25	5330.82	5330.56	5326.90	5330.32	5320.88	5330.67	5329.35	5330.71	5324.32
	*K* = 30	5332.12	5330.49	5329.33	5331.52	5324.61	5331.05	5330.97	5331.76	5327.56
Image 2	*K* = 15	2481.66	2481.76	2475.41	2480.13	2467.28	2481.16	2478.52	2481.33	2470.21
	*K* = 20	2486.29	2486.17	2481.53	2485.41	2475.05	2485.80	2484.23	2485.99	2478.01
	*K* = 25	2488.54	2488.31	2484.74	2487.95	2479.66	2488.01	2486.86	2488.35	2482.29
	*K* = 30	2489.44	2488.36	2486.81	2488.83	2482.40	2488.38	2488.37	2488.57	2484.96
Image 3	*K* = 15	771.60	771.62	767.93	770.55	762.37	770.83	768.89	771.28	763.82
	*K* = 20	773.75	773.75	770.98	773.46	766.32	773.27	771.86	773.50	768.12
	*K* = 25	774.93	774.85	772.55	774.63	769.19	774.41	773.50	774.58	770.63
	*K* = 30	775.28	774.84	773.76	775.13	770.19	774.28	774.37	774.81	772.03
Image 4	*K* = 15	1435.40	1435.50	1430.02	1433.67	1423.95	1434.72	1432.25	1435.20	1425.08
	*K* = 20	1438.74	1438.73	1434.77	1437.70	1429.61	1438.21	1436.56	1438.44	1431.47
	*K* = 25	1440.40	1440.32	1437.25	1439.89	1432.70	1440.14	1438.81	1439.96	1434.52
	*K* = 30	1440.94	1440.27	1438.89	1440.70	1434.50	1440.16	1439.70	1440.35	1437.04
Image 5	*K* = 15	1358.07	1357.88	1353.74	1356.55	1348.32	1357.48	1355.59	1357.86	1350.06
	*K* = 20	1360.28	1360.11	1357.01	1359.42	1352.25	1359.82	1358.53	1360.17	1354.20
	*K* = 25	1361.36	1361.13	1358.76	1360.73	1354.79	1360.97	1359.92	1361.13	1356.85
	*K* = 30	1361.42	1361.15	1359.94	1361.28	1356.65	1361.03	1360.83	1361.59	1358.38
Image 6	*K* = 15	5711.20	5711.24	5705.13	5709.50	5695.96	5711.09	5707.42	5711.17	5699.94
	*K* = 20	5716.32	5716.14	5711.59	5715.65	5704.46	5716.16	5713.75	5716.17	5708.04
	*K* = 25	5718.85	5718.63	5715.14	5718.50	5709.84	5718.81	5717.17	5718.66	5712.85
	*K* = 30	5720.02	5718.62	5717.44	5719.61	5712.58	5719.13	5718.62	5719.09	5715.69
Image 7	*K* = 15	4319.50	4319.26	4313.66	4317.80	4305.37	4318.93	4316.64	4319.19	4308.97
	*K* = 20	4323.90	4323.50	4319.36	4322.87	4312.75	4323.24	4322.08	4323.31	4316.47
	*K* = 25	4325.97	4325.58	4322.43	4325.35	4317.14	4325.33	4324.56	4325.68	4319.91
	*K* = 30	4326.86	4325.61	4324.20	4326.03	4320.13	4325.56	4325.76	4326.16	4322.58
Image 8	*K* = 15	2886.66	2887.16	2881.17	2885.41	2872.96	2886.40	2882.55	2886.21	2876.05
	*K* = 20	2891.25	2891.70	2887.24	2890.79	2880.48	2891.29	2889.15	2891.07	2884.26
	*K* = 25	2893.71	2893.86	2890.47	2893.46	2885.10	2893.64	2892.25	2893.36	2888.12
	*K* = 30	2894.79	2893.90	2892.49	2894.49	2887.98	2893.66	2893.59	2893.90	2890.83
Image 9	*K* = 15	4626.40	4626.45	4621.53	4624.94	4614.08	4625.91	4624.47	4626.19	4617.62
	*K* = 20	4631.01	4630.89	4627.26	4630.17	4621.58	4630.31	4629.18	4630.69	4624.51
	*K* = 25	4633.25	4633.10	4630.19	4632.88	4625.75	4632.59	4632.06	4632.87	4628.36
	*K* = 30	4634.35	4633.09	4632.21	4633.64	4628.15	4632.85	4633.30	4633.54	4630.76
Image 10	*K* = 15	3981.61	3982.44	3975.21	3980.50	3966.69	3981.77	3976.74	3981.14	3969.97
	*K* = 20	3987.37	3987.93	3982.76	3987.02	3975.57	3987.73	3984.93	3987.12	3978.43
	*K* = 25	3990.45	3990.69	3986.71	3990.32	3981.01	3990.61	3988.50	3990.28	3984.18
	*K* = 30	3991.82	3990.79	3989.19	3991.68	3983.91	3991.06	3990.49	3990.88	3987.45
Image 11	*K* = 15	3828.42	3828.32	3821.16	3826.54	3810.40	3828.36	3825.15	3828.04	3814.68
	*K* = 20	3835.61	3835.28	3829.65	3834.70	3821.41	3835.52	3833.28	3835.15	3825.75
	*K* = 25	3839.06	3838.73	3834.58	3838.59	3827.92	3838.89	3837.29	3838.71	3831.48
	*K* = 30	3840.72	3838.74	3837.43	3839.95	3831.76	3839.56	3839.41	3840.11	3835.34
Image 12	*K* = 15	2793.98	2793.62	2787.03	2791.88	2778.89	2793.43	2791.19	2793.58	2781.17
	*K* = 20	2798.83	2798.51	2793.47	2797.29	2786.70	2798.31	2796.67	2798.60	2790.04
	*K* = 25	2801.21	2800.82	2797.17	2800.32	2791.37	2800.93	2799.70	2801.03	2794.51
	*K* = 30	2802.09	2800.86	2799.28	2801.68	2794.30	2801.29	2801.16	2801.86	2797.28

**Table A3:** FSIM statistical results based on the Kapur entropy method.

Different Images	Metric	ACOADE	COA	DE	PSO	AOA	WOA	SCSO	GWO	MGO
Image 1	*K* = 15	0.9608	0.9542	0.8643	0.9582	0.9135	0.9570	0.9622	0.9572	0.9293
	*K* = 20	0.9758	0.9647	0.8953	0.9718	0.9339	0.9706	0.9750	0.9732	0.9465
	*K* = 25	0.9838	0.9738	0.9221	0.9807	0.9478	0.9781	0.9808	0.9816	0.9622
	*K* = 30	0.9884	0.9793	0.9355	0.9864	0.9563	0.9819	0.9856	0.9868	0.9695
Image 2	*K* = 15	0.9486	0.9391	0.8357	0.9416	0.8895	0.9397	0.9484	0.9422	0.9090
	*K* = 20	0.9698	0.9577	0.8751	0.9608	0.9154	0.9605	0.9683	0.9622	0.9306
	*K* = 25	0.9793	0.9687	0.9081	0.9768	0.9334	0.9720	0.9764	0.9756	0.9506
	*K* = 30	0.9846	0.9750	0.9236	0.9838	0.9456	0.9779	0.9814	0.9820	0.9590
Image 3	*K* = 15	0.9200	0.9196	0.7942	0.9172	0.8551	0.9105	0.9108	0.9181	0.8681
	*K* = 20	0.9497	0.9470	0.8274	0.9496	0.8794	0.9443	0.9414	0.9486	0.8987
	*K* = 25	0.9651	0.9613	0.8556	0.9671	0.8991	0.9590	0.9591	0.9653	0.9124
	*K* = 30	0.9749	0.9698	0.8798	0.9773	0.9137	0.9689	0.9690	0.9739	0.9322
Image 4	*K* = 15	0.9545	0.9510	0.8293	0.9514	0.8903	0.9500	0.9514	0.9516	0.9088
	*K* = 20	0.9714	0.9665	0.8728	0.9708	0.9148	0.9667	0.9680	0.9720	0.9326
	*K* = 25	0.9807	0.9766	0.8993	0.9806	0.9294	0.9751	0.9769	0.9802	0.9494
	*K* = 30	0.9854	0.9797	0.9205	0.9865	0.9477	0.9800	0.9823	0.9861	0.9600
Image 5	*K* = 15	0.9660	0.9563	0.8393	0.9596	0.8942	0.9532	0.9654	0.9624	0.9004
	*K* = 20	0.9802	0.9692	0.8698	0.9777	0.9183	0.9690	0.9769	0.9782	0.9345
	*K* = 25	0.9871	0.9747	0.8923	0.9855	0.9362	0.9775	0.9843	0.9858	0.9529
	*K* = 30	0.9900	0.9785	0.9173	0.9897	0.9443	0.9811	0.9884	0.9878	0.9594
Image 6	*K* = 15	0.9066	0.9082	0.7968	0.9076	0.8432	0.9076	0.8951	0.9068	0.8622
	*K* = 20	0.9454	0.9414	0.8295	0.9466	0.8756	0.9436	0.9332	0.9439	0.8950
	*K* = 25	0.9645	0.9588	0.8619	0.9659	0.8962	0.9617	0.9561	0.9619	0.9129
	*K* = 30	0.9756	0.9684	0.8781	0.9772	0.9110	0.9698	0.9686	0.9704	0.9275
Image 7	*K* = 15	0.9530	0.9375	0.8598	0.9421	0.9035	0.9368	0.9539	0.9453	0.9187
	*K* = 20	0.9729	0.9567	0.9023	0.9649	0.9281	0.9586	0.9705	0.9683	0.9403
	*K* = 25	0.9812	0.9666	0.9164	0.9768	0.9412	0.9707	0.9792	0.9790	0.9539
	*K* = 30	0.9861	0.9737	0.9348	0.9854	0.9514	0.9762	0.9834	0.9845	0.9648
Image 8	*K* = 15	0.9594	0.9506	0.8699	0.9526	0.9062	0.9521	0.9588	0.9523	0.9282
	*K* = 20	0.9743	0.9686	0.8944	0.9735	0.9306	0.9698	0.9712	0.9722	0.9472
	*K* = 25	0.9816	0.9772	0.9167	0.9825	0.9436	0.9778	0.9779	0.9803	0.9561
	*K* = 30	0.9856	0.9812	0.9348	0.9870	0.9545	0.9828	0.9826	0.9843	0.9660
Image 9	*K* = 15	0.9407	0.9354	0.8516	0.9399	0.8858	0.9321	0.9363	0.9388	0.9076
	*K* = 20	0.9593	0.9553	0.8834	0.9606	0.9144	0.9549	0.9544	0.9583	0.9246
	*K* = 25	0.9700	0.9665	0.8950	0.9724	0.9233	0.9650	0.9659	0.9690	0.9428
	*K* = 30	0.9770	0.9733	0.9176	0.9795	0.9401	0.9732	0.9733	0.9761	0.9555
Image 10	*K* = 15	0.9736	0.9708	0.8813	0.9748	0.9236	0.9738	0.9668	0.9685	0.9417
	*K* = 20	0.9838	0.9812	0.9096	0.9851	0.9455	0.9835	0.9796	0.9824	0.9581
	*K* = 25	0.9885	0.9864	0.9322	0.9900	0.9612	0.9871	0.9848	0.9886	0.9693
	*K* = 30	0.9911	0.9882	0.9493	0.9926	0.9677	0.9896	0.9879	0.9905	0.9763
Image 11	*K* = 15	0.9642	0.9599	0.8571	0.9625	0.9112	0.9614	0.9608	0.9618	0.9308
	*K* = 20	0.9782	0.9727	0.8937	0.9772	0.9313	0.9751	0.9754	0.9760	0.9472
	*K* = 25	0.9851	0.9795	0.9142	0.9858	0.9477	0.9819	0.9823	0.9837	0.9611
	*K* = 30	0.9893	0.9840	0.9335	0.9902	0.9571	0.9859	0.9864	0.9882	0.9673
Image 12	*K* = 15	0.9770	0.9688	0.8682	0.9734	0.9235	0.9702	0.9772	0.9729	0.9416
	*K* = 20	0.9868	0.9783	0.9058	0.9845	0.9466	0.9812	0.9860	0.9846	0.9597
	*K* = 25	0.9911	0.9841	0.9356	0.9903	0.9568	0.9853	0.9902	0.9900	0.9702
	*K* = 30	0.9933	0.9866	0.9473	0.9932	0.9670	0.9883	0.9924	0.9925	0.9771

**Table A4:** PSNR statistical results based on the Kapur entropy method.

Different Images	Metric	ACOADE	COA	DE	PSO	AOA	WOA	SCSO	GWO	MGO
Image 1	*K* = 15	29.1657	28.1592	23.6648	28.6929	25.8114	28.4047	29.6044	28.4899	27.1300
	*K* = 20	31.6597	29.6623	25.5052	30.8372	27.5440	30.3769	31.7745	30.9279	28.8791
	*K* = 25	33.7493	31.4284	27.1371	32.8323	28.8166	32.0260	33.2505	32.9219	30.7393
	*K* = 30	35.4486	32.6358	28.4820	34.7572	30.0438	33.2404	34.8117	34.4900	31.8603
Image 2	*K* = 15	29.2612	28.1730	22.5261	28.5928	25.0613	28.1674	29.0220	28.3999	26.4718
	*K* = 20	32.1559	30.4375	24.9601	31.0281	26.9743	30.5979	31.6714	31.0495	28.4849
	*K* = 25	33.8900	32.2094	25.9121	33.5968	28.5689	32.3434	33.3331	32.9244	30.5401
	*K* = 30	35.4485	33.5391	28.2936	35.3106	29.7637	33.7873	34.7019	34.3881	31.4786
Image 3	*K* = 15	30.6888	30.2471	23.7093	30.5132	26.6128	29.6693	30.0567	30.1628	27.7295
	*K* = 20	32.9939	32.3314	25.6552	32.9945	28.1511	32.0876	32.3432	32.3570	29.7752
	*K* = 25	34.6291	33.8767	27.3297	34.7918	29.4894	33.5862	34.0193	34.1054	30.8423
	*K* = 30	36.0473	34.9641	28.8810	36.3238	31.1815	34.8580	35.4175	35.3548	32.3825
Image 4	*K* = 15	30.3536	29.5145	23.3122	30.0325	25.6003	29.4992	30.0496	29.6243	27.2002
	*K* = 20	32.5468	31.5023	25.1981	32.3508	27.2808	31.4693	32.1082	32.0660	29.1812
	*K* = 25	34.3314	33.2356	26.8437	34.1588	28.6473	32.9795	33.7450	33.6632	30.8576
	*K* = 30	35.6454	34.2474	28.6487	35.7078	30.5087	34.2208	34.9888	35.1752	32.2161
Image 5	*K* = 15	31.6270	30.3235	25.0446	30.6472	27.3377	30.0678	31.8635	30.7362	28.1077
	*K* = 20	33.9186	32.1265	26.8106	33.1397	28.9420	31.9586	33.8328	33.0928	30.2610
	*K* = 25	35.8836	33.2302	28.2986	34.9748	30.2484	33.6460	35.6092	35.0232	31.9006
	*K* = 30	37.1370	34.4156	29.9266	36.4818	30.9499	34.6656	37.0305	36.3623	32.7737
Image 6	*K* = 15	29.6864	28.7816	23.2823	29.1966	25.6676	28.6592	29.2321	28.8888	27.0741
	*K* = 20	32.2559	30.8323	25.0769	31.8945	27.4326	31.1763	31.4846	31.5115	28.9447
	*K* = 25	34.0679	32.3584	26.9583	33.8326	28.7713	32.7888	33.3525	33.3500	30.3929
	*K* = 30	35.5620	33.6847	28.4914	35.5375	29.9820	34.0035	34.6999	34.6233	31.6406
Image 7	*K* = 15	28.6020	27.0950	23.0687	27.7088	25.2156	27.2031	28.7986	27.8865	26.4319
	*K* = 20	31.7707	29.7308	25.2747	30.7411	27.1702	30.0914	31.1937	31.0014	28.3722
	*K* = 25	33.7947	31.2355	26.7131	32.9646	28.4100	31.7957	33.3578	33.1830	30.1213
	*K* = 30	35.3154	32.7106	28.5118	35.2399	29.6107	33.1379	34.3389	34.6129	31.6929
Image 8	*K* = 15	29.4145	28.4340	22.4722	28.9681	25.0292	28.5963	29.1621	28.5826	26.7175
	*K* = 20	31.6820	30.9731	24.8026	31.7729	27.0972	31.0564	30.9381	31.2504	28.7564
	*K* = 25	33.2322	32.6363	26.3560	33.8797	28.3286	32.6071	32.5270	32.9568	30.0954
	*K* = 30	34.6124	33.9674	28.0561	35.3373	29.7977	33.8503	33.8110	34.4028	31.6198
Image 9	*K* = 15	28.4674	28.4962	22.6454	28.5862	25.5520	28.3612	27.8213	28.2714	26.2948
	*K* = 20	31.1093	30.9484	24.9925	31.6613	27.0507	30.7561	29.8136	31.0487	28.5127
	*K* = 25	33.0519	32.2940	26.9066	33.5074	28.1056	32.2209	31.8696	32.6258	30.1525
	*K* = 30	34.5357	33.6549	27.9173	35.1445	29.6110	33.6638	33.4201	34.0374	31.5905
Image 10	*K* = 15	28.7275	28.6959	22.7249	29.5013	24.7254	29.1380	27.2447	28.5770	26.4355
	*K* = 20	31.1027	31.2018	24.6105	32.1236	26.8902	31.5893	29.6632	31.2649	28.4690
	*K* = 25	32.7213	32.9767	26.4947	34.1149	28.7642	33.2493	31.3480	33.1777	30.1812
	*K* = 30	34.0151	34.0220	28.1065	35.7144	29.9070	34.4192	32.5861	34.3848	31.5645
Image 11	*K* = 15	29.7143	28.8759	23.0195	29.4689	25.4055	28.9975	29.3695	29.0990	27.1012
	*K* = 20	32.0108	30.8731	24.9212	31.7492	27.0655	31.0652	31.5309	31.2708	28.6958
	*K* = 25	33.8034	32.3012	26.4151	33.7916	28.6285	32.5398	33.1180	33.0277	30.3870
	*K* = 30	35.2473	33.5574	28.0640	35.3888	29.8731	33.8353	34.6289	34.5231	31.5028
Image 12	*K* = 15	30.1687	29.0167	23.4634	29.6149	25.7644	29.0337	30.1469	29.3329	27.1404
	*K* = 20	32.5459	30.9828	25.6287	32.1075	27.6814	31.2708	32.4482	31.7826	29.1918
	*K* = 25	34.4509	32.5238	27.6058	34.1266	28.8127	32.7110	34.1385	33.7499	30.6317
	*K* = 30	35.8611	33.5763	28.6656	35.7533	30.1738	34.0049	35.5522	35.1572	32.0211

**Table A5:** SSIM statistical results based on the Kapur entropy method.

Different Images	Metric	ACOADE	COA	DE	PSO	AOA	WOA	SCSO	GWO	MGO
Image 1	*K* = 15	0.7978	0.7520	0.7140	0.7702	0.7311	0.7795	0.8692	0.7769	0.7729
	*K* = 20	0.8870	0.7933	0.7697	0.8399	0.7949	0.8307	0.9180	0.8692	0.8298
	*K* = 25	0.9405	0.8558	0.8014	0.8922	0.8291	0.8786	0.9352	0.9198	0.8882
	*K* = 30	0.9647	0.8808	0.8443	0.9308	0.8617	0.9164	0.9627	0.9452	0.9049
Image 2	*K* = 15	0.9178	0.9016	0.7882	0.9058	0.8459	0.9027	0.9168	0.9071	0.8721
	*K* = 20	0.9452	0.9297	0.8414	0.9336	0.8808	0.9328	0.9424	0.9379	0.9019
	*K* = 25	0.9583	0.9490	0.8767	0.9554	0.9040	0.9487	0.9546	0.9541	0.9259
	*K* = 30	0.9680	0.9574	0.8966	0.9662	0.9198	0.9595	0.9632	0.9648	0.9366
Image 3	*K* = 15	0.8969	0.8969	0.7703	0.8941	0.8366	0.8868	0.8883	0.8951	0.8495
	*K* = 20	0.9278	0.9263	0.8074	0.9279	0.8608	0.9225	0.9183	0.9273	0.8816
	*K* = 25	0.9456	0.9439	0.8398	0.9472	0.8816	0.9403	0.9389	0.9468	0.8965
	*K* = 30	0.9577	0.9546	0.8584	0.9600	0.9082	0.9526	0.9511	0.9576	0.9171
Image 4	*K* = 15	0.9253	0.9220	0.8005	0.9221	0.8584	0.9202	0.9222	0.9218	0.8826
	*K* = 20	0.9470	0.9427	0.8428	0.9459	0.8870	0.9417	0.9443	0.9481	0.9091
	*K* = 25	0.9610	0.9578	0.8641	0.9597	0.9052	0.9552	0.9563	0.9612	0.9286
	*K* = 30	0.9688	0.9636	0.8961	0.9694	0.9280	0.9625	0.9646	0.9705	0.9415
Image 5	*K* = 15	0.8876	0.8302	0.6618	0.8453	0.7522	0.8307	0.9122	0.8603	0.7632
	*K* = 20	0.9376	0.8675	0.7377	0.9099	0.7855	0.8794	0.9402	0.9179	0.8535
	*K* = 25	0.9606	0.8992	0.7922	0.9397	0.8382	0.9137	0.9589	0.9465	0.8819
	*K* = 30	0.9695	0.9128	0.8219	0.9577	0.8525	0.9261	0.9689	0.9607	0.8956
Image 6	*K* = 15	0.8664	0.8309	0.7132	0.8348	0.7695	0.8360	0.8687	0.8454	0.8097
	*K* = 20	0.9189	0.8855	0.7748	0.9030	0.8135	0.8891	0.9132	0.9076	0.8525
	*K* = 25	0.9425	0.9077	0.8135	0.9323	0.8478	0.9188	0.9371	0.9364	0.8825
	*K* = 30	0.9572	0.9326	0.8438	0.9509	0.8737	0.9364	0.9515	0.9502	0.9009
Image 7	*K* = 15	0.8918	0.8351	0.7498	0.8647	0.8031	0.8533	0.8945	0.8749	0.8390
	*K* = 20	0.9424	0.8984	0.8364	0.9181	0.8557	0.9087	0.9360	0.9295	0.8824
	*K* = 25	0.9595	0.9219	0.8528	0.9448	0.8794	0.9359	0.9570	0.9550	0.9134
	*K* = 30	0.9691	0.9378	0.8882	0.9665	0.9035	0.9449	0.9657	0.9650	0.9355
Image 8	*K* = 15	0.9249	0.9179	0.7848	0.9158	0.8470	0.9160	0.9216	0.9155	0.8783
	*K* = 20	0.9508	0.9455	0.8346	0.9487	0.8841	0.9447	0.9451	0.9480	0.9094
	*K* = 25	0.9650	0.9598	0.8676	0.9654	0.9052	0.9586	0.9598	0.9628	0.9248
	*K* = 30	0.9737	0.9684	0.8965	0.9739	0.9218	0.9666	0.9687	0.9711	0.9414
Image 9	*K* = 15	0.9301	0.9185	0.8361	0.9281	0.8766	0.9161	0.9285	0.9238	0.8989
	*K* = 20	0.9460	0.9376	0.8768	0.9442	0.9035	0.9376	0.9431	0.9424	0.9109
	*K* = 25	0.9557	0.9494	0.8844	0.9557	0.9097	0.9463	0.9540	0.9524	0.9299
	*K* = 30	0.9638	0.9568	0.9014	0.9644	0.9272	0.9563	0.9609	0.9598	0.9431
Image 10	*K* = 15	0.9538	0.9522	0.8426	0.9554	0.8933	0.9543	0.9442	0.9497	0.9150
	*K* = 20	0.9698	0.9686	0.8733	0.9718	0.9223	0.9706	0.9633	0.9690	0.9385
	*K* = 25	0.9780	0.9767	0.9059	0.9805	0.9402	0.9777	0.9727	0.9787	0.9531
	*K* = 30	0.9829	0.9805	0.9192	0.9856	0.9513	0.9820	0.9783	0.9826	0.9628
Image 11	*K* = 15	0.9380	0.9192	0.8025	0.9267	0.8638	0.9250	0.9421	0.9293	0.8942
	*K* = 20	0.9642	0.9465	0.8501	0.9551	0.8956	0.9520	0.9631	0.9580	0.9208
	*K* = 25	0.9750	0.9590	0.8820	0.9720	0.9204	0.9638	0.9727	0.9708	0.9425
	*K* = 30	0.9814	0.9670	0.9095	0.9805	0.9351	0.9716	0.9791	0.9790	0.9519
Image 12	*K* = 15	0.9285	0.8883	0.7611	0.9042	0.8328	0.9010	0.9404	0.9121	0.8658
	*K* = 20	0.9636	0.9292	0.8364	0.9464	0.8818	0.9364	0.9649	0.9519	0.9105
	*K* = 25	0.9759	0.9509	0.8790	0.9688	0.9013	0.9501	0.9767	0.9709	0.9309
	*K* = 30	0.9830	0.9556	0.9026	0.9794	0.9208	0.9640	0.9826	0.9793	0.9471

**Table A6:** FSIM statistical results based on the Otsu method.

Different Images	Metric	ACOADE	COA	DE	PSO	AOA	WOA	SCSO	GWO	MGO
Image 1	*K* = 15	0.9688	0.9662	0.8687	0.9652	0.9312	0.9674	0.9655	0.9677	0.9396
	*K* = 20	0.9792	0.9764	0.8958	0.9769	0.9489	0.9781	0.9776	0.9790	0.9432
	*K* = 25	0.9851	0.9822	0.9157	0.9832	0.9596	0.9836	0.9840	0.9843	0.9565
	*K* = 30	0.9885	0.9806	0.9413	0.9849	0.9660	0.9826	0.9868	0.9855	0.9663
Image 2	*K* = 15	0.9493	0.9378	0.8456	0.9357	0.9056	0.9428	0.9552	0.9417	0.9204
	*K* = 20	0.9697	0.9543	0.8849	0.9552	0.9246	0.9640	0.9709	0.9619	0.9432
	*K* = 25	0.9801	0.9675	0.9046	0.9665	0.9396	0.9732	0.9802	0.9740	0.9565
	*K* = 30	0.9864	0.9705	0.9232	0.9724	0.9531	0.9752	0.9832	0.9784	0.9663
Image 3	*K* = 15	0.9571	0.9477	0.8001	0.9422	0.8920	0.9527	0.9555	0.9484	0.9116
	*K* = 20	0.9765	0.9661	0.8443	0.9626	0.9156	0.9707	0.9730	0.9674	0.9363
	*K* = 25	0.9858	0.9753	0.8729	0.9719	0.9360	0.9783	0.9813	0.9776	0.9519
	*K* = 30	0.9884	0.9769	0.8868	0.9772	0.9461	0.9785	0.9844	0.9818	0.9623
Image 4	*K* = 15	0.9641	0.9531	0.8428	0.9478	0.9221	0.9555	0.9641	0.9526	0.9310
	*K* = 20	0.9803	0.9693	0.8820	0.9656	0.9346	0.9734	0.9787	0.9713	0.9509
	*K* = 25	0.9886	0.9785	0.9057	0.9762	0.9498	0.9836	0.9865	0.9809	0.9624
	*K* = 30	0.9912	0.9792	0.9197	0.9821	0.9594	0.9833	0.9885	0.9840	0.9724
Image 5	*K* = 15	0.9820	0.9768	0.8249	0.9737	0.9399	0.9785	0.9759	0.9784	0.9485
	*K* = 20	0.9894	0.9854	0.8719	0.9846	0.9553	0.9861	0.9846	0.9872	0.9622
	*K* = 25	0.9927	0.9892	0.9026	0.9892	0.9624	0.9900	0.9889	0.9904	0.9720
	*K* = 30	0.9930	0.9881	0.9241	0.9907	0.9699	0.9895	0.9912	0.9919	0.9788
Image 6	*K* = 15	0.9311	0.9290	0.7947	0.9227	0.8636	0.9296	0.9170	0.9302	0.8802
	*K* = 20	0.9584	0.9536	0.8271	0.9538	0.8948	0.9556	0.9487	0.9556	0.9094
	*K* = 25	0.9727	0.9671	0.8617	0.9684	0.9173	0.9682	0.9664	0.9699	0.9325
	*K* = 30	0.9792	0.9632	0.8899	0.9728	0.9274	0.9683	0.9734	0.9703	0.9459
Image 7	*K* = 15	0.9596	0.9539	0.8675	0.9541	0.9247	0.9568	0.9605	0.9569	0.9349
	*K* = 20	0.9728	0.9662	0.9070	0.9676	0.9398	0.9697	0.9749	0.9711	0.9529
	*K* = 25	0.9800	0.9724	0.9272	0.9739	0.9522	0.9771	0.9819	0.9794	0.9634
	*K* = 30	0.9863	0.9727	0.9383	0.9790	0.9607	0.9774	0.9855	0.9820	0.9712
Image 8	*K* = 15	0.9468	0.9380	0.8763	0.9350	0.9140	0.9414	0.9535	0.9383	0.9293
	*K* = 20	0.9673	0.9549	0.8974	0.9506	0.9324	0.9594	0.9712	0.9545	0.9460
	*K* = 25	0.9793	0.9649	0.9279	0.9621	0.9415	0.9700	0.9807	0.9691	0.9603
	*K* = 30	0.9844	0.9708	0.9369	0.9684	0.9563	0.9742	0.9832	0.9768	0.9680
Image 9	*K* = 15	0.9514	0.9482	0.8592	0.9456	0.9081	0.9472	0.9468	0.9491	0.9190
	*K* = 20	0.9684	0.9657	0.8799	0.9649	0.9296	0.9639	0.9627	0.9659	0.9409
	*K* = 25	0.9780	0.9753	0.9014	0.9765	0.9440	0.9732	0.9740	0.9750	0.9545
	*K* = 30	0.9831	0.9734	0.9217	0.9784	0.9524	0.9724	0.9789	0.9772	0.9638
Image 10	*K* = 15	0.9509	0.9408	0.8758	0.9314	0.9157	0.9410	0.9641	0.9364	0.9367
	*K* = 20	0.9745	0.9606	0.9093	0.9515	0.9358	0.9654	0.9788	0.9633	0.9574
	*K* = 25	0.9826	0.9735	0.9384	0.9656	0.9545	0.9753	0.9861	0.9743	0.9672
	*K* = 30	0.9898	0.9768	0.9499	0.9752	0.9665	0.9795	0.9888	0.9827	0.9764
Image 11	*K* = 15	0.9640	0.9614	0.8617	0.9597	0.9279	0.9624	0.9595	0.9622	0.9355
	*K* = 20	0.9779	0.9750	0.8958	0.9753	0.9454	0.9762	0.9741	0.9763	0.9538
	*K* = 25	0.9850	0.9817	0.9214	0.9834	0.9563	0.9825	0.9817	0.9836	0.9645
	*K* = 30	0.9885	0.9797	0.9383	0.9851	0.9630	0.9827	0.9858	0.9855	0.9723
Image 12	*K* = 15	0.9817	0.9796	0.8782	0.9784	0.9551	0.9802	0.9794	0.9804	0.9589
	*K* = 20	0.9891	0.9858	0.9137	0.9854	0.9659	0.9864	0.9866	0.9874	0.9714
	*K* = 25	0.9928	0.9890	0.9337	0.9895	0.9725	0.9896	0.9908	0.9909	0.9783
	*K* = 30	0.9939	0.9872	0.9552	0.9913	0.9761	0.9896	0.9924	0.9915	0.9831

**Table A7:** PSNR statistical results based on the Otsu method.

Different Images	Metric	ACOADE	COA	DE	PSO	AOA	WOA	SCSO	GWO	MGO
Image 1	*K* = 15	30.3843	29.7086	24.0619	29.8521	26.8780	29.8179	30.0062	29.8462	27.8288
	*K* = 20	32.5978	31.4833	25.6337	31.7787	28.4145	31.7376	32.1434	31.8947	29.7611
	*K* = 25	34.4344	32.9228	26.8485	33.4187	29.7050	33.1750	33.8926	33.4439	31.4052
	*K* = 30	35.6375	33.0110	28.9007	34.2543	30.8221	33.4991	34.9931	34.1603	32.7880
Image 2	*K* = 15	30.2407	28.6731	22.9566	28.7753	26.1166	29.0945	30.5491	29.0326	27.6639
	*K* = 20	33.0871	30.7115	25.4453	31.1685	27.7124	31.7313	32.8648	31.5736	29.7611
	*K* = 25	35.1410	32.6767	26.8950	32.9655	29.1141	33.3544	34.6635	33.5563	31.4052
	*K* = 30	36.6151	33.3562	28.5398	34.1793	30.6971	33.9473	35.7687	34.5372	32.7880
Image 3	*K* = 15	33.0543	30.8385	24.1847	30.4576	27.6497	32.0337	33.5111	31.1122	30.0403
	*K* = 20	36.3577	33.5678	26.6366	33.1504	29.1996	34.6983	35.7565	34.0669	32.0911
	*K* = 25	38.6775	35.5689	28.4729	34.8934	31.3220	36.1953	37.3478	36.1906	33.7769
	*K* = 30	39.7077	36.4789	29.4393	36.5519	32.7978	36.6888	38.3881	37.5548	35.1625
Image 4	*K* = 15	31.4357	29.5405	23.5290	29.2774	27.3310	29.9538	31.6050	29.5657	28.6121
	*K* = 20	34.3224	31.9986	25.8109	31.6624	28.2868	32.6636	33.9455	32.3877	30.6588
	*K* = 25	36.6455	33.8554	27.5072	33.5946	29.9602	34.9203	35.7961	34.5144	32.2607
	*K* = 30	37.8258	34.4993	28.5798	35.3556	31.3562	35.2731	36.6967	35.6660	33.8470
Image 5	*K* = 15	35.7630	34.4808	25.0911	34.3668	30.4411	34.4590	34.3899	34.7504	31.4454
	*K* = 20	38.1548	36.5743	27.1712	36.7948	31.7860	36.5125	36.4616	36.9841	33.0033
	*K* = 25	39.9101	38.0964	28.8614	38.6694	32.6431	38.0284	37.9095	38.4070	34.5297
	*K* = 30	40.0113	38.1762	30.1117	39.7253	33.7851	38.1618	39.2187	39.3819	35.7700
Image 6	*K* = 15	30.4420	29.1591	22.9341	29.3321	26.1433	29.3512	30.0365	29.5221	27.7245
	*K* = 20	32.9970	31.1960	24.9157	31.7789	28.0445	31.4064	32.2203	31.8014	29.4753
	*K* = 25	34.9483	32.8563	27.2461	33.4905	29.4710	33.0455	34.1408	33.7467	31.3234
	*K* = 30	36.1001	33.1293	28.9542	34.3934	30.7583	33.6256	35.2897	34.3611	32.6953
Image 7	*K* = 15	29.6142	28.4640	23.4300	28.7723	26.2253	28.9408	29.9732	28.8797	27.4192
	*K* = 20	32.3434	30.6768	25.8466	31.1704	27.6575	31.1024	32.5056	31.4424	29.6323
	*K* = 25	34.2258	32.1087	27.5013	32.8385	29.0580	33.0899	34.2830	33.4730	31.0068
	*K* = 30	36.1156	32.3951	28.7417	34.1960	30.5679	33.4035	35.6086	34.3928	32.7196
Image 8	*K* = 15	28.1438	26.6318	23.1445	26.4163	24.8828	27.1728	28.6265	26.7460	26.6777
	*K* = 20	31.2486	29.2105	24.8717	28.7921	26.8169	30.0106	31.5369	29.3275	28.8478
	*K* = 25	33.6197	31.1875	27.3388	30.8294	27.9412	32.1004	33.5482	32.0150	30.9675
	*K* = 30	35.1006	32.6591	28.3926	32.4600	30.2144	33.0947	34.6657	33.6962	32.3549
Image 9	*K* = 15	30.8923	30.3825	23.2234	30.3812	27.1708	30.2664	30.2714	30.4295	28.2966
	*K* = 20	33.3555	32.6149	25.3693	32.8664	29.0365	32.4050	32.4757	32.6875	30.4337
	*K* = 25	35.2488	34.3217	26.8529	34.9797	30.4735	33.9299	34.3818	34.3463	32.0389
	*K* = 30	36.6034	34.4493	28.7622	35.7572	31.5009	34.2019	35.5997	35.0119	33.4650
Image 10	*K* = 15	27.3755	26.2174	22.4100	25.4297	24.0947	26.2631	28.1500	25.7708	26.0880
	*K* = 20	30.5295	29.0571	24.5447	28.0057	26.0475	29.5859	30.6990	29.2962	28.6587
	*K* = 25	32.4714	31.3983	26.9529	30.2880	28.2842	31.6533	32.4317	31.4963	30.2561
	*K* = 30	34.2346	32.2495	28.2272	32.3874	29.7942	32.6502	33.6571	33.0871	31.9466
Image 11	*K* = 15	30.0077	29.3690	23.0794	29.4610	26.5286	29.4211	29.4208	29.4090	27.4072
	*K* = 20	32.3332	31.4561	25.0597	31.8676	28.1049	31.6163	31.6034	31.6053	29.3589
	*K* = 25	34.1569	33.0296	26.8210	33.7549	29.4034	33.2150	33.3150	33.3279	30.8363
	*K* = 30	35.4001	33.0424	28.4714	34.6506	30.4969	33.6638	34.5367	34.1918	32.2282
Image 12	*K* = 15	31.8454	31.0820	23.9401	31.1469	27.9237	31.1346	31.2492	31.1876	28.6510
	*K* = 20	34.2560	33.0602	26.0467	33.1708	29.3367	33.1093	33.2517	33.3192	30.5129
	*K* = 25	36.0264	34.5312	27.4976	35.0079	30.4804	34.7231	34.9624	35.0318	31.9794
	*K* = 30	36.7953	34.4141	29.4646	36.3070	31.3522	35.0224	36.0481	35.8398	33.1593

**Table A8:** SSIM statistical results based on the Otsu method.

Different Images	Metric	ACOADE	COA	DE	PSO	AOA	WOA	SCSO	GWO	MGO
Image 1	*K* = 15	0.8693	0.8452	0.7396	0.8550	0.7714	0.8535	0.8981	0.8603	0.8053
	*K* = 20	0.9140	0.8750	0.7842	0.8879	0.8238	0.8915	0.9322	0.9028	0.9160
	*K* = 25	0.9445	0.8991	0.8233	0.9067	0.8608	0.9118	0.9570	0.9229	0.9345
	*K* = 30	0.9645	0.9076	0.8589	0.9179	0.8918	0.9171	0.9630	0.9321	0.9476
Image 2	*K* = 15	0.9248	0.9063	0.8047	0.9047	0.8590	0.9127	0.9317	0.9120	0.8853
	*K* = 20	0.9539	0.9322	0.8554	0.9344	0.8886	0.9447	0.9535	0.9424	0.9160
	*K* = 25	0.9679	0.9519	0.8760	0.9516	0.9101	0.9589	0.9661	0.9602	0.9345
	*K* = 30	0.9754	0.9564	0.9003	0.9607	0.9287	0.9618	0.9715	0.9663	0.9476
Image 3	*K* = 15	0.9524	0.9432	0.7793	0.9380	0.8805	0.9453	0.9458	0.9440	0.9011
	*K* = 20	0.9709	0.9634	0.8294	0.9618	0.9066	0.9645	0.9634	0.9645	0.9266
	*K* = 25	0.9803	0.9734	0.8586	0.9721	0.9277	0.9735	0.9729	0.9749	0.9432
	*K* = 30	0.9831	0.9737	0.8741	0.9773	0.9374	0.9725	0.9777	0.9778	0.9545
Image 4	*K* = 15	0.9507	0.9402	0.8071	0.9335	0.9004	0.9407	0.9467	0.9394	0.9092
	*K* = 20	0.9694	0.9603	0.8535	0.9566	0.9155	0.9628	0.9646	0.9623	0.9337
	*K* = 25	0.9788	0.9715	0.8828	0.9700	0.9334	0.9752	0.9743	0.9732	0.9466
	*K* = 30	0.9824	0.9717	0.8992	0.9766	0.9440	0.9747	0.9781	0.9768	0.9591
Image 5	*K* = 15	0.9694	0.9584	0.6959	0.9569	0.9017	0.9593	0.9617	0.9617	0.9075
	*K* = 20	0.9815	0.9731	0.7513	0.9742	0.9236	0.9735	0.9758	0.9767	0.9346
	*K* = 25	0.9869	0.9802	0.8090	0.9829	0.9334	0.9810	0.9820	0.9833	0.9499
	*K* = 30	0.9868	0.9798	0.8449	0.9861	0.9440	0.9805	0.9857	0.9861	0.9613
Image 6	*K* = 15	0.8789	0.8445	0.7142	0.8455	0.7634	0.8532	0.8861	0.8596	0.8135
	*K* = 20	0.9257	0.8912	0.7836	0.8992	0.8164	0.8966	0.9209	0.9073	0.8569
	*K* = 25	0.9493	0.9196	0.8223	0.9249	0.8559	0.9234	0.9458	0.9383	0.8951
	*K* = 30	0.9604	0.9231	0.8478	0.9358	0.8833	0.9313	0.9563	0.9436	0.9165
Image 7	*K* = 15	0.8926	0.8523	0.7796	0.8691	0.8091	0.8760	0.9168	0.8727	0.8515
	*K* = 20	0.9391	0.9058	0.8320	0.9151	0.8519	0.9150	0.9499	0.9281	0.8991
	*K* = 25	0.9552	0.9260	0.8735	0.9373	0.8843	0.9474	0.9627	0.9524	0.9186
	*K* = 30	0.9699	0.9289	0.8940	0.9538	0.9140	0.9472	0.9700	0.9605	0.9456
Image 8	*K* = 15	0.9304	0.9142	0.8098	0.9092	0.8669	0.9193	0.9349	0.9150	0.8938
	*K* = 20	0.9582	0.9438	0.8409	0.9390	0.8986	0.9495	0.9596	0.9437	0.9221
	*K* = 25	0.9730	0.9583	0.8850	0.9564	0.9167	0.9642	0.9722	0.9634	0.9438
	*K* = 30	0.9793	0.9647	0.8987	0.9650	0.9379	0.9676	0.9762	0.9717	0.9550
Image 9	*K* = 15	0.9379	0.9294	0.8586	0.9294	0.8935	0.9284	0.9338	0.9316	0.9062
	*K* = 20	0.9574	0.9522	0.8740	0.9521	0.9152	0.9495	0.9503	0.9534	0.9278
	*K* = 25	0.9694	0.9641	0.8944	0.9670	0.9317	0.9616	0.9636	0.9651	0.9421
	*K* = 30	0.9755	0.9640	0.9116	0.9705	0.9408	0.9628	0.9699	0.9686	0.9523
Image 10	*K* = 15	0.9372	0.9254	0.8371	0.9153	0.8836	0.9256	0.9444	0.9197	0.9123
	*K* = 20	0.9641	0.9514	0.8778	0.9430	0.9125	0.9559	0.9654	0.9534	0.9389
	*K* = 25	0.9753	0.9668	0.9132	0.9606	0.9370	0.9688	0.9758	0.9675	0.9543
	*K* = 30	0.9835	0.9700	0.9294	0.9709	0.9514	0.9729	0.9809	0.9757	0.9653
Image 11	*K* = 15	0.9409	0.9255	0.8064	0.9274	0.8841	0.9293	0.9419	0.9319	0.8993
	*K* = 20	0.9637	0.9491	0.8566	0.9532	0.9131	0.9542	0.9623	0.9570	0.9291
	*K* = 25	0.9761	0.9625	0.8907	0.9679	0.9297	0.9655	0.9736	0.9703	0.9449
	*K* = 30	0.9816	0.9608	0.9123	0.9729	0.9440	0.9694	0.9781	0.9740	0.9580
Image 12	*K* = 15	0.9500	0.9360	0.7921	0.9386	0.8916	0.9425	0.9559	0.9424	0.9056
	*K* = 20	0.9500	0.9360	0.7921	0.9386	0.8916	0.9425	0.9559	0.9424	0.9348
	*K* = 25	0.9837	0.9668	0.8766	0.9699	0.9339	0.9701	0.9813	0.9763	0.9494
	*K* = 30	0.9858	0.9611	0.9154	0.9778	0.9432	0.9725	0.9841	0.9804	0.9611
